# E50A Mutation Increases the Bioluminescence Activity of picALuc

**DOI:** 10.3390/bios16030167

**Published:** 2026-03-17

**Authors:** Kabir H. Biswas

**Affiliations:** College of Health and Life Sciences, Hamad Bin Khalifa University, Doha, Qatar; kbiswas@hbku.edu.qa

**Keywords:** ALuc, bioluminescence, luciferase, molecular dynamics simulation, picALuc

## Abstract

A miniaturized variant of the artificial luciferase (ALuc), named picALuc, has been generated through the deletion of N- and C-terminal residues in ALuc. Although picALuc is small and active, questions remain regarding its the structural organization and inter-residue interactions in the protein. Here, combining computational analysis and mutational studies, we show that the E50A mutation in picALuc results in an increased bioluminescence activity of the protein. Specifically, we generated a structural model of picALuc using the available structure of the *Gaussia* luciferase (GLuc) that revealed a ‘hole’ in the structure due to the deletion of N-terminal α-helices. Gaussian-accelerated molecular dynamics (GaMD) simulation revealed a rapid ‘compaction’ of the picALuc structure during the initial phase of the simulation and a number of residues such as E10, E50, and D94 showed salt bridge interactions. Mutation of the residues E10, E50, and D94 individually to an A revealed increased bioluminescence activity of the E50A mutant, while E10A and D94A mutants showed activities similar to the WT protein in living cells. In vitro assays revealed an increase in the *V*_max_ of the E50A mutant, while *K*_half_ and thermal stability of the mutant remained unchanged. Further, dynamic cross-correlation and principal component analyses of the GaMD simulation trajectories of the WT and the E50A mutant picALuc revealed altered collective dynamics in the protein. Finally, we developed a protein fragment complementation assay using picALuc that allows for the monitoring protein–protein interactions (PPIs) in live cells. We envisage that the brighter picALuc reported here will find broad applicability in developing bioluminescence-based assays.

## 1. Introduction

Bioluminescence has been utilized extensively in developing a wide range of biological applications, including monitoring gene expression, protein–protein interaction (PPI), and protein structural changes [1,2,3,4,5,6,7,8,9]. These applications are primarily based on the activity of luciferase proteins that emit light at a characteristic wavelength upon the oxidation of a cognate substrate, with or without requiring ATP. To further increase their utility in biological and biomedical applications, several luciferase variants have been characterized or developed with a range of spectral (e.g. different the peak emission wavelength), biophysical (e.g., increased thermal stability), and bioluminescence, (e.g., increased photon emission), properties. Some of these include *Renilla* luciferase (RLuc) variants [10,11,12,13,14], such as Rluc8 [15] and Super Rluc8 [16], and the generation of the NanoLuc (NLuc) luciferase from the deep-sea shrimp (*Oplophorus gracilirostris*) luciferase [17,18,19,20].

Efforts have been made to generate artificial luciferases (ALucs) using the consensus amino acid sequence of *Copepod* luciferases [21], leading to the generation of a number of ALucs with unique sequence and properties such as enhanced and stable bioluminescence. The ALucs were then utilized for the development of assays such as mammalian two-hybrid assay, live cell imaging and bioluminescent antibodies. Additional efforts toward generating ALucs using the same approach resulted in the generation of brighter variants with a preference for native coelenterazine as a substrate and some with altered spectral properties [22]. Additionally, analysis of amino acid sequences of the ALucs revealed a role for the C-terminal residues in bioluminescence activity of the ALucs but not in their spectral properties. The latest addition in this series of luciferases is a miniaturized variant of one of the ALucs (ALuc30) generated previously [23]. Aptly referred to as picALuc due to its small size with a molecular weight of 13 kDa, it was generated through the deletion of residues 1–54 at the N-terminal and residues 175–194 at the C-terminal of ALuc30. Importantly, picALuc was reported to show thermal stability and brightness similar to that of NLuc, thus raising the possibility of its utilization in a wider range of bioluminescence-based assays, including PPI and Bioluminescence Resonance Energy Transfer (BRET) [23,24,25,26,27,28]. However, the impact of the deletion of the N- and C-terminal residues on the structure and residue level interactions in picALuc are not well understood.

In the current study, we generated a structural model of picALuc using the available structure of *Gaussia* luciferase (Gluc) [29] as a template, which revealed a large ‘hole’ in the N-terminal part of the protein due to the absence of α-helices 1 and 2 that were originally present in the template structure. We then performed an all-atom, explicit solvent, 1 μs long Gaussian accelerated MD (GaMD) simulation to understand structural dynamics and inter-residue interactions in the protein. The simulation revealed structural compaction of the protein early on during the simulation, largely due to the movement of the N-terminal α-helices of picALuc into the positions occupied by α-helices that were present in ALuc but deleted in picALuc. Additionally, we observed increased number of salt bridge interactions in the protein and mutation of salt bridge interaction forming residues E10, E50, and D94 to A revealed a large increase in the bioluminescence activity of picALuc with the E50A mutation. Biochemical and biophysical characterization revealed that the increased bioluminescence activity of the E50A mutant is due an increase in the *V*_max_ of the protein without any significant alteration in *K*_half_ or thermal stability of the protein. We then performed GaMD simulation of the E50A mutant picALuc and compared it with that of the WT protein which revealed differences in the collective dynamics in the protein, in addition to a much reduced PC1 contribution of the A50 residue in the E50A mutant picALuc compared to E50 in the WT protein. Finally, we report the development of a picALuc-based protein fragment complementation assay that allows monitoring PPI in live cells.

## 2. Methods

### 2.1. Structural Modeling of WT and E50A Mutant picALuc

We utilized the available nuclear magnetic resonance (NMR)-derived structure of GLuc (PDB: 7D2O) [29] to generate a homology-based structural model of picALuc using the SWISS-MODEL webserver (https://swissmodel.expasy.org/interactive, accessed on 20 December 2025) [30,31,32], which typically uses the first conformer when the template structure contains an ensemble of conformers such as in the case of NMR-derived structures. The two proteins, picALuc and GLuc, show considerable sequence conservation (92.5% similarity; and 85% identity) with key differences in the N- and C-terminal (Figure S1A), allowing for the high confidence structural modeling of picALuc. Notably, picALuc lacks N-terminal α-helices 1 and 2, and some residues in the C-terminal region of ALuc (Figure S1B). A structural model of picALuc was also generated using Alpha-Fold2 [33]. The structural model of ALuc was also generated using the SWISS-MODEL webserver (https://swissmodel.expasy.org/interactive) [30,31,32]. The quality of the modeled structure was assessed using the MolProbity software (version 4.4) [34] available as a part of the SWISS-MODEL webserver (https://swissmodel.expasy.org/interactive) [30,31,32] with a score of 2.90 and with only two Gly residues (located in the long loop between α-helices 3 and 4) out of a total of 120 residues (1.69%; the GLuc template structure 7D2O [29] showed 4 out of 168 residues, which is equal to 2.8%) as Ramachandran plot outliers. Additionally, all disulfide bridges observed in the GLuc structure were maintained in the modeled picALuc structure (Figure S1C). The structural model of the E50A mutant picALuc was generated by the backbone rotamer-dependent replacement of the residue E with an A using PyMOL (The PyMOL Molecular Graphics System, Version 2.0.0, Schrödinger, LLC; pymol.org; New York, NY, USA).

### 2.2. MD Simulations

GaMD simulations of the modeled WT and E50A mutant picALuc and ALuc structures were performed essentially as described previously [24,32]. Input files, including topology and parameter files, were prepared using the QwikMD plugin [35] available in the Visual Molecular Dynamics (VMD) software (version 1.9.3) [36], and simulations were performed using the Nanoscale Molecular Dynamics (NAMD) software (version 2.13) [37] and CHARMM36 force field [38]. Briefly, the proteins were solvated in explicit solvent using a TIP3P [39] cubic water box containing 0.15 M NaCl for charge neutralization and periodic boundary conditions applied with a 2 fs integration time-step. Prior to production simulations, energy minimization was performed on each system for 2000 steps (4 ps) [32]. Subsequently, the systems were gradually heated from 60 K to 310 K at intervals of 1 K to reach the 310 K equilibrium temperature. Following thermalization, temperature was maintained at 310 K using Langevin temperature control and pressure was maintained at 1.0 atm using Nose–Hoover Langevin piston pressure control [40]. The systems were then equilibrated for 500,000 steps (1 ns). GaMD simulations were then run using the integrated GaMD module in the NAMD software (version 2.13) and its default parameters [41,42,43]. This included 2 ns of conventional molecular dynamics equilibration run for collecting potential statistics that were used for calculating acceleration parameters, and another 50 ns equilibration run with the addition of boost potential [42,44], and finally GaMD production runs for 1 μs. A 400 ps preparatory run was included before each of the equilibration steps in GaMD. All GaMD simulations were run at the ‘dual-boost’ level with one boost potential applied to the dihedral energy term and the other applied to the total potential energy term [41,43,45] with a standard deviation upper limit set to 6.0 kcal/mol. Short-range non-bonded interactions were defined at a 12 Å cutoff with 10 Å switching distance, while Particle-Mesh Ewald (PME) scheme was used to handle long-range electrostatic interactions at 1 Å PME grid spacing. Simulation frames were saved every 10,000 steps (20 ps). Detailed protocol used for the GaMD simulations are included in the Supplementary Materials Text. Simulation trajectories were analyzed using available tools in the VMD software (version 1.9.3) [36] and the MDAnalysis package (version 2.0) [46,47]. Backbone root-mean-square deviation (RMSD), root-mean-square fluctuation (RMSF), solvent-accessible surface area (SASA), radius of gyration (RoG), energy calculations, secondary structure salt bridges, and H-bond occupancy were performed using the VMD software (version 1.9.3) [36,48,49]. Pairwise RMSD, number of H-bonds formed and S atom distances in disulfide bridge forming residues were calculated using the MDAnalysis package (version 2.0) [46,47]. The dynamic cross-correlation (DCC) analysis of the trajectories were performed using the calc_correlation.py script available as a part of the MD-TASK suite (version 1.0.1) of MD trajectory analysis software [50] either over the whole 1 μs trajectory with a step size of 2 (alternating frames) or the last 900 ns of the trajectory. Principal component analysis (PCA) was performed either over the whole 1 μs trajectory or the last 900 ns of the trajectory using the PCA module available in the MDAnalysis package (version 2.0; version 2.9.0 for the last 900 ns trajectory analysis) [46,47]. Contribution of individual residues to PC1 and 2 were calculated by projecting the principal components on Cα atoms of the proteins using modules in the MDAnalysis package (version 2.0; version 2.9.0 for the last 900 ns trajectory analysis) [46,47] followed by calculation of individual Cα atom displacement (root mean squared). The conventional MD simulation of picALuc was performed using input files generated using the QwikMD plug-in [35] available in the VMD software (version 1.9.3) [36] and using the NAMD software (version 2.13) [37] and CHARMM36 force field [38]. Movies were prepared from 500 frames out of the 50,000 frames of the 1 μs trajectory (with a step size of 100 frames) generated using the VMD Movie Maker plug-in (version 1.9.3) [36] and compiled at 20 fps using the Fiji distribution of ImageJ software (version 1.53v) [51]. Clustering was performed using the k-means clustering algorithm available as a part of the MDAnalysis package (version 2.0) [46,47]. All structural images were generated using PyMOL (The PyMOL Molecular Graphics System, Version 2.0.0, Schrödinger, LLC; pymol.org; New York, NY, USA).

### 2.3. mGreenLantern (mGL)-picALuc Plasmid Construct Generation

The complete nucleotide and amino acid sequences of all constructs designed and utilized in the current manuscript have been included in Supplementary Materials Text. A codon optimized gene sequence of picALuc generated using the previously described amino acid sequence of the protein [23] was synthesized and subcloned into the pcDNA3.1-mGL-NLuc plasmid (which was generated by synthesizing the mGL gene and subcloned into the previously described pmNG-M^pro^-Nter-auto-NLuc plasmid using *Eco*RI-*Bam*HI sites) using *Bam*HI and *Xho*I restriction enzyme sites (GenScript, Singapore) to generate the pcDNA3.1-mGL-picALuc plasmid. Additionally, the nucleotide sequences coding for the SARS-CoV-2 M^pro^ N-terminal autocleavage sequence was included in the N-terminal [24,52] and 3×FLAG-tag sequence was included at the C-terminal of the picALuc sequence. Glu10Ala (E10A), Glu50Ala (E50A), and Asp94Ala (D94A) mutations were generated in the pcDNA3.1-mGL-picALuc plasmid (GenScript, Singapore). For generating the picSm and picSm-GCN4 fragment expressing plasmids, nucleotide sequences encoding picALuc N-terminal α-helix 1 (K1 to A22) or one with the GCN4 peptide (EELLSKNYHLENEVARLKK) were synthesized and subcloned into the pcDNA3.1-mGL-NLuc plasmid using *Bam*HI-*Xho*I sites (GenScript, Singapore). For generating the picLg- and picLg-GCN4 fragment expressing plasmids, nucleotide sequences encoding picALuc residues G23-C120 or one with the GCN4 peptide were synthesized and subcloned into pcDNA3.1-mGL-NLuc plasmid using the *Hind*III-*Xho*I sites (GenScript, Singapore).

### 2.4. Cell Culture and Transfection

HEK 293T cells were cultured in Dulbecco’s Modified Eagle Medium (DMEM) supplemented with 10% fetal bovine serum, and 1% penicillin–streptomycin and grown at 37 °C in 5% CO_2_ [27]. Transfections were performed using polyethyleneimine (PEI) transfection reagent according to the manufacturer’s protocol. Briefly, HEK 293T cells were seeded onto 96-well white plates 24 h prior to transfection. Plasmid DNA (encoding either the WT or mutant picALuc luciferases; 125 ng/well), Opti-MEM (Invitrogen, Carlsbad, CA, USA; 31985088; 25 μL/well) and PEI lipid (Sigma-Aldrich, St. Louis, MO, USA; 408727-100 mL; 0.625 μL/well) were mixed and incubated at room temperature for 30 min prior to addition to the cells. For in vitro biochemical and thermal stability assays, cells were transfected in 60 mm dishes using appropriate proportion of DNA, PEI lipid and Opti-MEM. For the protein fragment complementation assays, HEK 293T cells were transfected with a range of picSm or picSm-GCN4 and picLg or picLg-GCN4 plasmid DNA ratios with picLg or picLg-GCN4 maintained at 25 ng/well while total plasmid concentrations were maintained at 150 ng/well using a control plasmid (pcDNA3-HA). The PEI stock solution of 2 mg/mL was prepared by diluting in sterile Milli-Q water and stored at −80 °C for subsequent usage.

### 2.5. Live Cell Assays

Live cell assays to determine fluorescence, bioluminescence and bioluminescence emission spectra were performed by transfecting HEK 293T cells with either the WT or the mutant pcDNA3.1-mGL-picALuc plasmid constructs. For SARS-CoV-2 M^pro^-mediated cleavage of mGL-M^pro^-Nter-picALuc assays, cells transfected with pcDNA3.1-mGL-picALuc plasmid along with either pLVX-EF1alpha-SARS-CoV-2-nsp5-2xStrep-IRES-Puro (M^pro^ WT) (a gift from Nevan Krogan (Addgene plasmid # 141370; http://n2t.net/addgene:141370 (accessed on 1 June 2020); RRID:Addgene_141370) [53] or pLVX-EF1alpha-SARS-CoV-2-nsp5-C145A-2xStrep-IRES-Puro (C145A mutant M^pro^) plasmid (a gift from Nevan Krogan (Addgene plasmid # 141371; http://n2t.net/addgene:141371 (accessed on 1 June 2020); RRID:Addgene_141371) [53]. For the picALuc-based protein complementation assays, cells were transfected with the indicated ratios of plasmid DNA amounts. After 48 h of transfection, mGL fluorescence was measured using a Tecan SPARK^®^ multimode microplate reader (Tecan, Mannedorf, Switzerland) prior to bioluminescence measurements. Samples were excited at a wavelength 480 nm and emission acquired at a wavelength of 530 nm. Bioluminescence was measured in the cells using a Tecan SPARK^®^ multimode microplate reader after addition of luciferase substrate coelenterazine h at a final concentration of 5 μM. Bioluminescence spectra was acquired using the Tecan SPARK^®^ multimode microplate reader between 380 to 665 nm wavelengths with an acquisition time of 400 ms for each wavelength. Bioluminescence spectral data was normalized by dividing emissions at all wavelengths with that of emission at 483 nm. BRET was calculated as a ratio of emission at 516 nm (corresponding to the emission maxima of acceptor mGL) and 483 nm (corresponding to the emission maxima of donor picALuc) [24,25,26,27,31,54,55,56,57,58,59,60,61]. Bioluminescence of each sample was calculated from emission intensities between 380 and 665 nm and was first normalized with mGL fluorescence (relative to WT values) and then with that of bioluminescence of WT picALuc. For protein complementation assay, total bioluminescence (wavelengths between 380 and 665 nm) was measured in all samples following mGL fluorescence measurements. All experiments were performed thrice in triplicates.

### 2.6. In Vitro Assays

In vitro assays were performed using lysates prepared from cells expressing the respective picALuc constructs as described previously [25,26,27,31,54,55,56,57,58,59,60,61]. After 48 h of transfection, cells were washed thrice in chilled Dulbecco’s phosphate-buffered saline (DPBS) and harvested and lysed by sonication on ice in a buffer containing 50 mM HEPES (pH 7.5), 100 mM NaCl, 2 mM EDTA, 1 mM dithiothreitol (DTT), 1× protease inhibitor cocktail (ThermoFisher Scientific, Waltham, MA, USA) and 10% glycerol. Sonicated samples were centrifuged at 4 °C for 1 h and supernatant was collected for further experiments. For biochemical experiments to determine *K*_half_ (substrate affinity) and *V*_max_ (reaction velocity), equivalent amounts of the proteins were taken after normalization with mGL fluorescence and incubated with a range of substrate, coelenterazine h, concentrations. Data were fit to an allosteric sigmoidal model considering bioluminescence output (counts per second) representing the rate of reaction to determine *V*_max_ and *K*_half_ values. Thermal stability of the proteins was determined by measuring bioluminescence from equivalent amounts of cell lysates after incubation at a range of temperatures for 10 min. Data were fit to a Boltzmann sigmoidal model to determine the temperature at which the protein shows half maximal activity (*T*_m_). Bioluminescence was measured after addition of the substrate in a GloMax^®^ Discover Microplate Reader (Promega, Madison, WI, USA). All experiments were performed thrice in triplicates.

### 2.7. Data Analysis and Figure Preparation

MATLAB (release 2021b), Matplotlib (version 3.7 and later), GraphPad Prism (version 9 for macOS, GraphPad Software, La Jolla, CA, USA; www.graphpad.com) and Microsoft Excel were used for data analysis and graph preparation. Figures were assembled using Adobe Illustrator (version 27.0 and later).

## 3. Results and Discussion

### 3.1. Molecular Dynamics Simulation Reveals picALuc Structural Features and Reorganization

**Figure 1:**
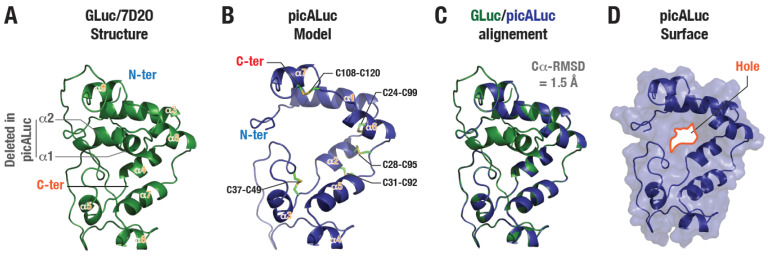
picALuc sequence and structural features. (**A**) Cartoon representation of GLuc structure highlighting various secondary structural elements (α-helices), disulfide bridges and the equivalent N-terminal region deleted in picALuc. (**B**) Cartoon representation of picALuc structural model generated using the available GLuc structure (PDB: 7D2O) [29]. Disulfide bridges, secondary structural elements (α-helices) and N and C-termini. (**C**) Cartoon representation of aligned GLuc and modeled picALuc structures. (**D**) Cartoon and surface representation of the modeled picALuc structure showing the presence of a ‘hole’ due to the absence of the N-terminal region (α-helices 1 and α2 of GLuc).

Deletion mutagenesis of ALuc, a synthetically designed luciferase protein, led to the generation of the small, 13 kDa, luciferase, picALuc (Figure S1A) [23]. Specifically, the authors deleted residues 1 to 54 at the N-terminal (comprising of α-helices 1 and 2) and residues 175 to 194 at the C-terminal side (comprising of an extended unstructured loop region) (Figure S1B). To better understand the structural features of picALuc and especially how deletion of such structural segments of the original ALuc protein might impact the structure, we generated a structural model of the protein using the available NMR structure of GLuc (PDB: 7D2O; Figure S2) [29]. For this, the amino acid sequences of the picALuc and GLuc were aligned (Figure S1A) and a structural model was generated using the SWISS-MODEL webserver [30,31,32] (Figure 1A–D). One of the key features of GLuc is the presence of five disulfide bridges formed by residue pairs C28-C95, C31-C92, C37-C49, C24-C99 and C108-C120 (Figure 1A and Figure S1C), which were maintained in the generated picALuc model. Additionally, all secondary structural elements were maintained in the modeled structure of picALuc (Figure 1A,B), except for those which were deleted in picALuc (α-helices in Figure 1B were numbered according to the picALuc sequence). A structural model of picALuc generated using Alpha-Fold2 also showed similar secondary structural elements and their organization (RMSD between SWISS-MODEL and Alpha-Fold2 generated structural models = 3.276 A; Figure S3). Overall, GLuc and the modeled picALuc structures showed a Cα atom RMSD value of 1.5 Å (Figure 1C). However, a closer inspection of the two structures revealed that the modeled picALuc structure to be non-compact containing a ‘hole’ at the N-terminal side. This is likely due to the absence of the α-helices 1 and 2 in the picALuc sequence (deleted in picALuc) but are present in the GLuc structure (Figure 1D).

**Figure 2:**
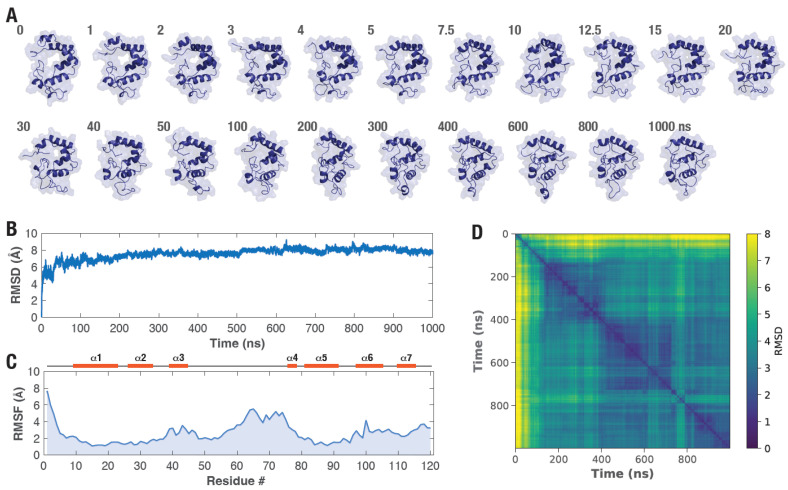
GaMD simulation reveals structural reorganization in picALuc. (**A**) Cartoon and surface representation of picALuc at the indicated period over the course of 1 μs GaMD simulation showing rapid structural evolution of the protein. Note that the frames shown are to best capture the structural during the initial phase of the simulation and the time difference is not uniform. Numbers shown are GaMD simulation time in ns. (**B**,**C**) Graphs showing Cα atom RMSD (**B**) and RMSF (**C**) values of picALuc obtained from the GaMD simulation. Secondary structural elements (α-helices) are highlighted in the RMSD graph for reference. (**D**) Heat map showing pairwise RMSD of Cα atom of picALuc. Note the significant changes in the initial phases of the simulation. RMSD values are in Å.

To determine if the modeled picALuc structure is stable or assumes a more compact form through conformational rearrangement, we performed molecular dynamics (MD) simulations with the modeled structure of picALuc, an approach that has been found useful for other luciferases such as NLuc and GLuc [62,63,64,65,66,67,68]. For this, we set up an all atom, explicit solvent MD simulation and ran the simulation for 1 μs using the NAMD software (version 2.13) [37]. We employed GaMD simulation, which is a robust method to enhance the conformational sampling of proteins through the addition of a harmonic boost potential to smoothen the proteins potential energy surface [43], to better explore the conformational dynamics of the protein with the possibility of reaching a stable, ‘native-like’ conformation within the simulation period of 1 μs (Supplementary Video S1) [41,42]. Analysis of the GaMD simulation trajectory revealed a rapid compaction of the picALuc structure within the first 10 ns of the simulation with further changes observed until 50 ns (Figure 2A). Root-mean-square deviation (RMSD) of Cα atom distances revealed large changes in the initial phases of the simulation with stabilization of the RMSD trace observed after about 100 ns (Figure 2B). Additionally, the root mean square fluctuation (RMSF) analysis of the trajectory revealed largest fluctuations in residues corresponding to the loop between α-helices 3 and 4 while residues corresponding to α-helix 3 in the N-terminal side and α-helices 6 and 7 in the C-terminal side (Figure 2C). Further, the pairwise RMSD analysis of the trajectory also captured the large structural changes in picALuc in the initial 100 ns of the simulation with largest changes seen in the first 10 ns of the simulation (Figure 2D).

**Figure 3:**
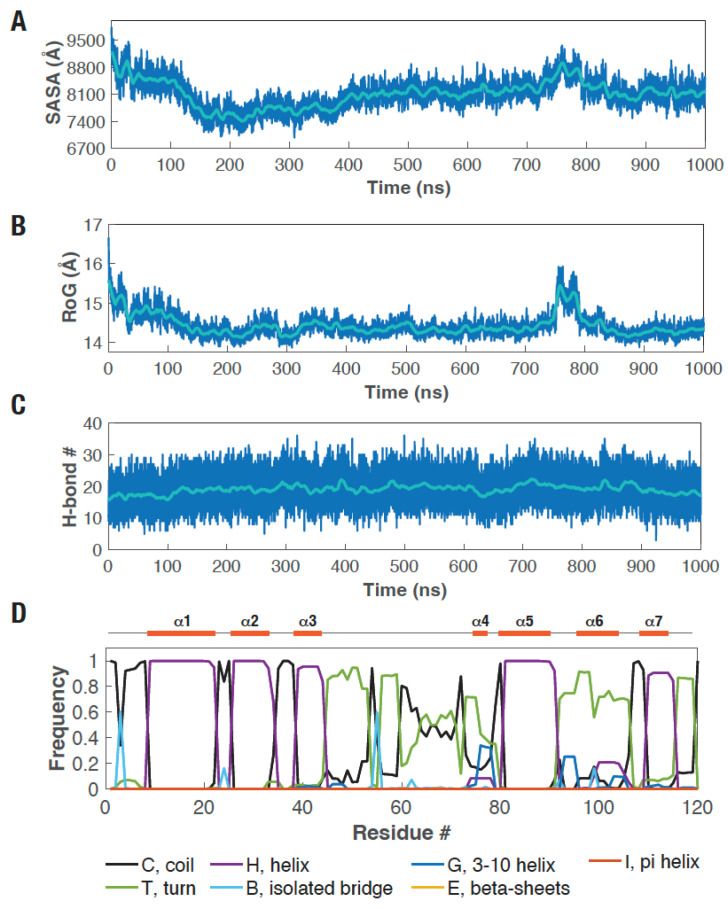
Structural features of picALuc. (**A**–**C**) Graphs showing solvent accessible surface area (SASA) (**A**), radius of gyration (RoG) (**B**), and number of H-bonds (**C**) over the course of 1 μs GaMD simulation. (**D**) Graph showing the frequency of various secondary structural elements in picALuc over the course of 1 μs GaMD simulation. Schematic above shows the location of various α-helices in the picALuc structural model used for the GaMD simulation.

We then performed solvent accessible surface area (SASA) and radius of gyration (RoG) analysis of the protein to confirm the structural compaction, which revealed a rapid shift in the conformational ensemble of the protein during the simulation (Figure 3A,B). The number of hydrogen bonds (H-bonds) remained similar during the simulation (Figure 3C; Table S1), suggesting no large changes between the picALuc conformers. Additionally, secondary structure analysis revealed changes only in the C-terminal part of the loop between α-helices 3 and 4 and residues in the C-terminal, short α-helix 6 exhibiting largely as a turn and only about 20% of the simulation time as the modeled helical structure (Figure 3D; Figure S4). These results indicate that the structural compaction of picALuc observed during the simulation is likely due to movement of the initially modeled α-helices. We note that all five disulfide bridges were maintained all throughout the simulation (Figure S5).

### 3.2. Increased Number of Salt Bridge Interactions Formed in picALuc

**Figure 4:**
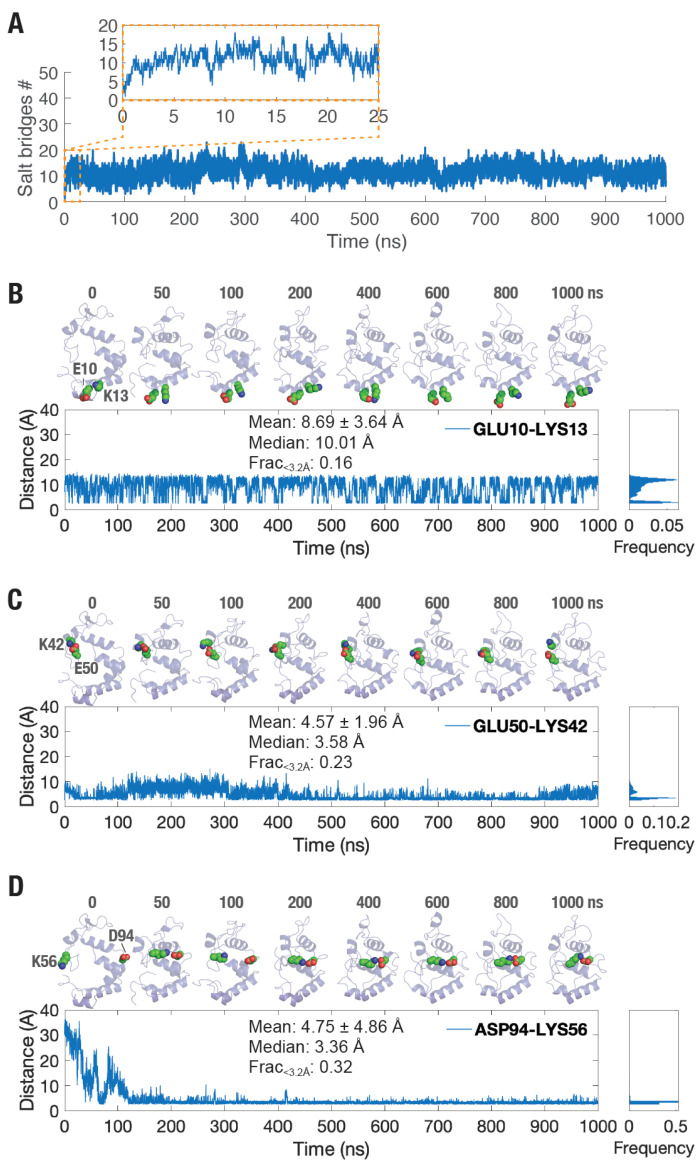
Salt bridge interactions in picALuc. (**A**) Graph showing number of salt bridges formed during a 1 μs long GaMD simulation. Inset shows increase in the number of salt bridges during the early phases of the simulation (0–25 ns). (**B**–**D**) Cartoon representation (top panels) and graphs showing inter-residue distances (bottom panels) of salt bridge forming residue pairs Glu10 and Lys13 (**B**), Glu50 and Lys42 (**C**), and Asp94 and Lys56 (**D**). The mean and median distances and fractional occupancies of each of the salt bridges are shown as insets and numbers on top indicate the GaMD simulation time in ns. Note that the picALuc structure has been reoriented to best show the salt bridge interactions and the side chains of the salt bridge forming residues have been highlighted using spheres with red color for carboxyl group (from negatively charged residues) and blue color for amine group (from positively charged residues).

Given the observation of increased electrostatic interaction (Figure 3D) and the presence of a large number of charged residues in picALuc (Figure S1B), we next examined the salt bridge interactions formed during the simulation. Determining the total number of salt bridge interactions formed with an oxygen–nitrogen distance cutoff of 3.2 Å revealed an increase in the number of salt bridge interactions formed by the protein (Figure 4A), with largest increases observed during the initial phase of the simulation suggesting a shift in the conformational ensemble of the protein. We, therefore, analyzed the trajectory for salt bridge interactions and found several salt bridge interactions that were formed while a number of such interactions were lost across the simulation (Figure S6). In addition, several salt bridge interactions were found to be fluctuating or were formed transiently (Figure S7). For instance, residues E10 and K13 (both located in the α-helix 1) formed a salt bridge interaction that showed fluctuating distance between the two amino acid residues with a mean (±s.d.) and median distance of 8.69 ± 3.64 and 10.01 Å, respectively, and a 16% fractional occupancy below 3.2 Å (Figure 4B). Importantly, we observed a frequent salt bridge interactions formed between E16 and R26, E50 and K36 and E50 and K42 throughout the trajectory. For instance, residues E50 (located in the loop between α-helices 3 and 4) and K42 (located in the α-helix 3) formed a salt bridge interaction with a mean (±s.d.) and median distance of 4.57 ± 1.96 and 3.58 Å, respectively and a 23% fractional occupancy below 3.2 Å (Figure 4C). Additionally, residues D94 (located in the loop between α-helices 5 and 6) and K56 (located in the loop between α-helices 3 and 4) frequently formed a salt bridge interaction across the simulation frames with a mean (±s.d.) and median distances of 4.75 ± 4.86 and 3.36 Å, respectively, and a 32% fractional occupancy below 3.2 Å (Figure 4D).

Overall, the GaMD simulations of picALuc revealed large-scale structural reorganization including dynamic changes in non-covalent interactions within the protein. To rule out the possibility that these structural changes are not an artefact of the GaMD simulations, we performed a 250 ns long, all atom, explicit solvent conventional MD simulations of the protein using the NAMD software (version 2.13) (Figure S8) [24,25,32,37,55,56,58,59,61]. The conventional MD simulation also revealed similar structural dynamics (Figure S8A–C) and changes in the structure of the protein including a large reduction in the radius of gyration of the protein during the initial stages of the simulation (Figure S8D) and secondary structure content (Figure S8E). Notably, the E50 residue showed frequent salt bridge interaction with the K42 residue (Figure S9). Further, to confirm if indeed these large changes in the picALuc structure are due to the lack of the N-terminal residue that are internal to the core of the protein domain and are deleted in picALuc (Figure S10A), we generated a structural model of ALuc, which showed the presence of the N- and C-terminal residues as seen with the GLuc structure (Figure S10A), and performed a 1 μs long GaMD simulation with the structural model of the ALuc protein that contains both N-terminal as well as C-terminal residues. C-terminal residues of ALuc that are deleted in picALuc are not part of the core of the protein and therefore, are less likely to impact the structural dynamics of the protein (Figure S10A). Analysis of the GaMD simulation trajectory revealed a relatively stable structure of the protein with the N-terminal residues maintaining their location internal to the protein domain core (Figure S10B) and relatively small decrease in RoG values and some increase in the number of salt bridge interactions (Figure S10). Overall, these results suggest that the large structural changes observed in the case of picALuc are indeed likely due to the lack of the N-terminal residues.

### 3.3. Mutational Analysis Reveals Increased Bioluminescence of picALuc

**Figure 5:**
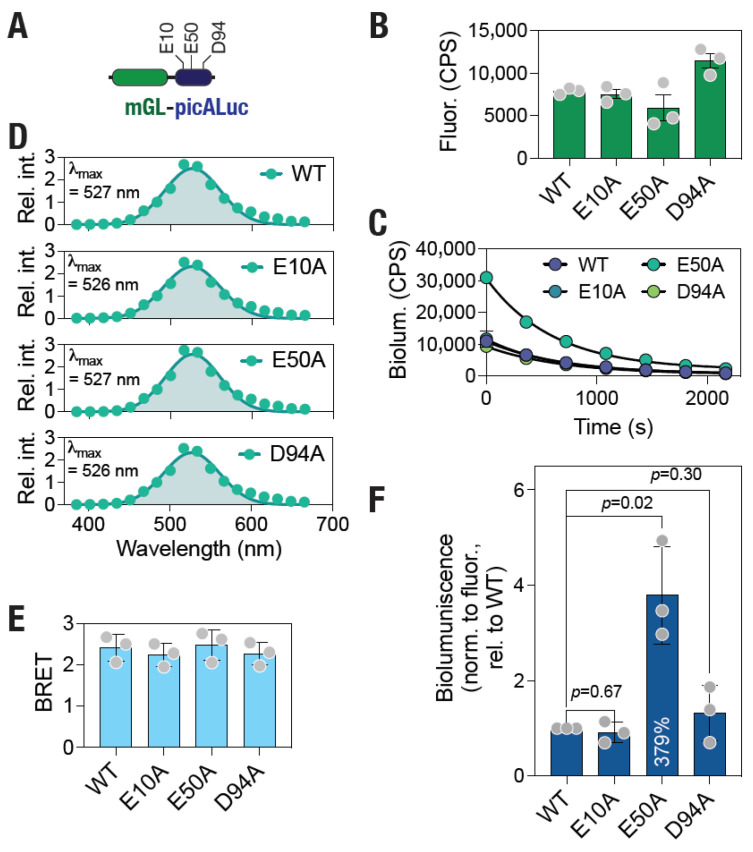
Increased bioluminescence of the E50A mutant picALuc. (**A**) Schematic showing the organization of the mGL-picALuc protein highlighting the relative location of E10, E50 and D94 residues in the protein. (**B**,**C**) Graphs showing fluorescence (**B**) and bioluminescence (**C**) determined from cells transfected with plasmid DNA for expressing either the WT or mutant mGL-picALuc proteins. Data shown are the mean ± s.e.m of measurements from a representative experiment performed in triplicates. (**D**) Graphs showing bioluminescence spectra of cells expressing either the WT or mutant mGL-picALuc proteins. Bioluminescence data were fit to a Gaussian distribution. (**E**) Graph showing BRET values of cells expressing either the WT or the mutant mGL-picALuc proteins. (**F**) Graph showing bioluminescence (normalized to mGL fluorescence values to account for differences in protein levels, either due to variation in cell density or transfection efficiency, and relative to the WT mGL-picALuc) of cells expressing either the WT or the mutant mGL-picALuc proteins. Data shown in E and F are the mean ± s.d. from three independent experiments performed in triplicates.

Following our observations with the specific salt bridge interactions above, we decided to determine their role in the bioluminescence activity of picALuc. For this, we mutated the residues E10, E50 and D94 to A (E10A, E50A and D94A, respectively) and expressed the proteins, along with the wild type (WT) protein, in mammalian (HEK 293T) cells. Additionally, we fused the mGL [69] green fluorescent protein at the N-terminal of picALuc to enable fluorescence-based monitoring of protein expression levels (Figure 5A). Fluorescence measurement of living cells transfected with plasmid DNA for expressing either the WT or the mutant picALuc proteins fused with the mGL [69] fluorescent protein showed similar levels of expression of the WT and E10A and E50A mutants while the D94A mutant showed a higher level of expression (Figure 5B). Bioluminescence measurements of cells expressing either the WT or the mutant picALuc proteins, on the other hand, showed similar activity of the WT and E10A and D94A mutant picALuc while the E50A mutant showed a higher activity (Figure 5C). Bioluminescence spectral measurements of the cells expressing the proteins showed a single peak at around 526 nm (Figure 5D) likely indicating a high efficiency of resonance energy transfer measured as a ratio of bioluminescence (Figure 5E). Indeed, inclusion of the self-cleaving P2A peptide [70,71] in between mGL and picALuc resulted in a blue-shifted spectrum and a significant decrease in BRET (Figure S11). Additionally, proteolytic cleavage of the SARS-CoV-2 M^pro^ N-terminal autocleavage site present in between mGL and picALuc upon co-expression of the WT, nut not the C145A mutant, M^pro^ protease resulted in shift in the spectra and a significant decrease in the BRET (Figure S12) [24,25,26,27,31,54,55,56,57,58,59,60,61]. Overall, we observed that the E50A mutant picALuc showed about 3.8 times bioluminescence activity (after normalization with total protein levels as determined from mGL fluorescence, which is fused to each of the picALuc proteins, to account for any variations in cell density, as well as efficiency of transfection of the plasmid DNA used for expression of the proteins) compared to the WT picALuc (Figure 5F). Thus, although the residue E50 formed a salt bridge interaction frequently during the GaMD simulation, mutation of the same resulted in an increased bioluminescence activity of picALuc.

### 3.4. E50A Mutation Results in Increased Enzymatic Activity Without Altering Thermal Stability of the Protein

**Figure 6:**
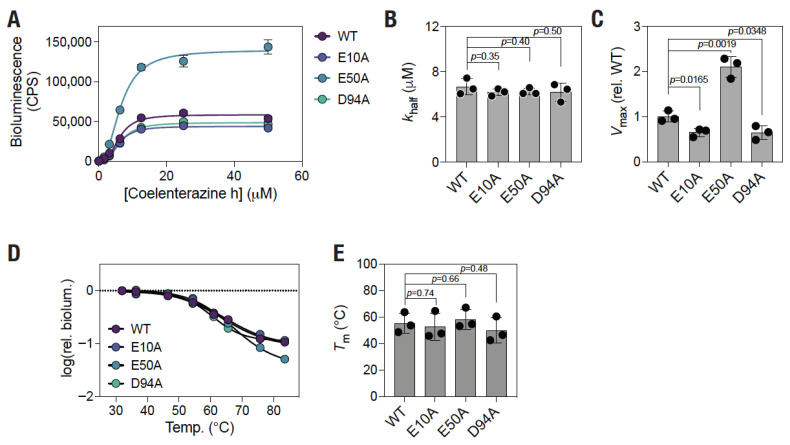
E50A mutation increases picALuc *V*_max_ without altering its thermal stability. (**A**) Graph showing bioluminescence activity in lysates prepared from cells expressing either the WT or mutant mGL-picALuc proteins under the indicated substrate (coelenterazine h) concentrations. Data shown are the mean ± s.e.m of measurements from a representative experiment and fit to an allosteric sigmoidal model. (**B**,**C**) Graphs showing *K*_half_ and *V*_max_ values of the WT and mutant mGL-picALuc proteins. Data shown are the mean ± s.d. from three independent experiments. (**D**) Graph showing relative bioluminescence activity of either the WT or the mutant mGL-picALuc protein incubated at the indicated temperatures. Dashed line shows the relative bioluminescence value of 1 at log(rel. biolum.) = 0. (**E**) Graph showing melting temperature of WT and mutant picALuc proteins obtained from Boltzmann sigmoidal model fitting of melting temperature curves. Data shown are the mean ± s.d. from three independent experiments.

Having observed an increased bioluminescence activity of the E50A mutant picALuc in living cells, we decided to determine the biochemical and thermal property of the protein in vitro. For this, HEK 293T cells transfected with the WT and various mutant picALuc proteins were used for preparing picALuc containing cell lysates to be used for in vitro assays. First, we performed enzyme kinetic studies with the WT and various mutants (with equivalent concentrations of proteins) under a range of substrate (coelenterazine h) concentrations and utilized the rate of photon emission as a measure of the enzymatic rate (bioluminescence; counts per second, CPS) and fitted the data to an allosteric sigmoidal model (Figure 6A). Consistent with the results obtained with live cells, we observed a higher bioluminescence activity with the E50A mutant picALuc compared to the WT picALuc. While the rate constant (*K*_half_) was found to be similar for all four proteins (*K*_half_ values of 6.6 ± 0.7, 6.2 ± 0.3, 6.2 ± 0.3 and 6.2 ± 0.8 μM for the WT, E10A, E50A and D94A mutant, respectively; Figure 6B), the maximum enzyme velocity (*V*_max_) was significantly higher for the E50A mutant compared to the WT picALuc (*V*_max_, relative to the WT of 1 ± 0.1, 0.6 ± 0.1, 2.1 ± 0.2 and 0.6 ± 0.2 for the WT, E10A, E50A and D94A mutant, respectively; Figure 6C). Additionally, we observed a significant decrease in the *V*_max_ of the E10A and D94A mutant compared to the WT picALuc (Figure 6C), suggesting a role for these residues and likely the salt bridge interactions formed by these residues in the catalytic activity of picALuc. Since these experiments were performed using lysates prepared from cells expressing the WT and the mutant picALuc proteins, it is not possible to directly determine the *k*_cat_ values of the proteins. However, given the increase in *V*_max_ but no significant change in the *K*_half_ values of the E50A mutant, as compared to the WT picALuc, at similar levels of the two proteins (normalized through mGL fluorescence values), it is likely that the E50A mutation has resulted in an increase in the *k*_cat_ of the enzyme, in a way similar to that reported for the D9R/K89R mutant of the NLuc luciferase protein [63].

Further, we determined the bioluminescence activity of the four picALuc proteins after incubation at a range of temperatures (from 32 to 84 °C) for 10 min. We fitted the data to a Boltzmann sigmoidal model to determine the melting temperature (*T*_m_) of the proteins (Figure 6D). We observed a relatively high thermal stability of picALuc with a *T*_m_ of 55.4 ± 7.7 °C (Figure 6E). However, no significant changes were observed in the *T*_m_ of the mutants, including E50A (Figure 6E). This likely reflects the thermal stability of the proteins is largely governed by the five disulfide bridges in the protein. Taken together, these data suggest that the E50A mutation increases the bioluminescence activity of picALuc by increasing its *V*_max_ without affecting its *K*_half_ or thermal stability.

We note that the increased bioluminescence activity of the E50A mutant picALuc, in comparison to the WT protein, appears to be largely in agreement with a previous report by Kim et al. [21] wherein authors analyzed sequences of a number of prototypical ALucs and showed that most of the ALucs contained an A residue at the position equivalent to E50 in picALuc. While such sequence conservation analyses are immensely useful in understanding sequence-function relationship of ALucs, our approach of combining structural dynamics analysis, including residue-residue interaction analysis and global correlated motions in picALuc, and functional analysis including biochemical and thermal stability analysis reported here provides further insights into mechanisms underlying picALuc activity. We further note that while our manuscript was published as a preprint in the biorxiv server [72] and was under review for publication as a peer reviewed article, Ohmuro-Matsuyama et al. [73] recapitulated results related to the increased activity of the E50A mutant picALuc reported here, and further extended these research efforts towards development of the picALuc2.0 protein, from the original picALuc1.0 reported previously, that showed much increased bioluminescence activity and contains a K, instead of A, residue at the E50 position. A closer inspection of ALuc sequences, as reported by Kim et al. [21], reveals that only one of the ALucs, out of several analyzed, contains a K in the position of E50 residue. Overall, it appears that picALuc, generated through deletion of parts of the ALuc luciferase, is a highly dynamic protein and approaches combining sequence, structure and functional analysis will likely lead to further improved variants of the protein in the future.

### 3.5. Altered Structural Dynamics in the E50A Mutant picALuc

**Figure 7:**
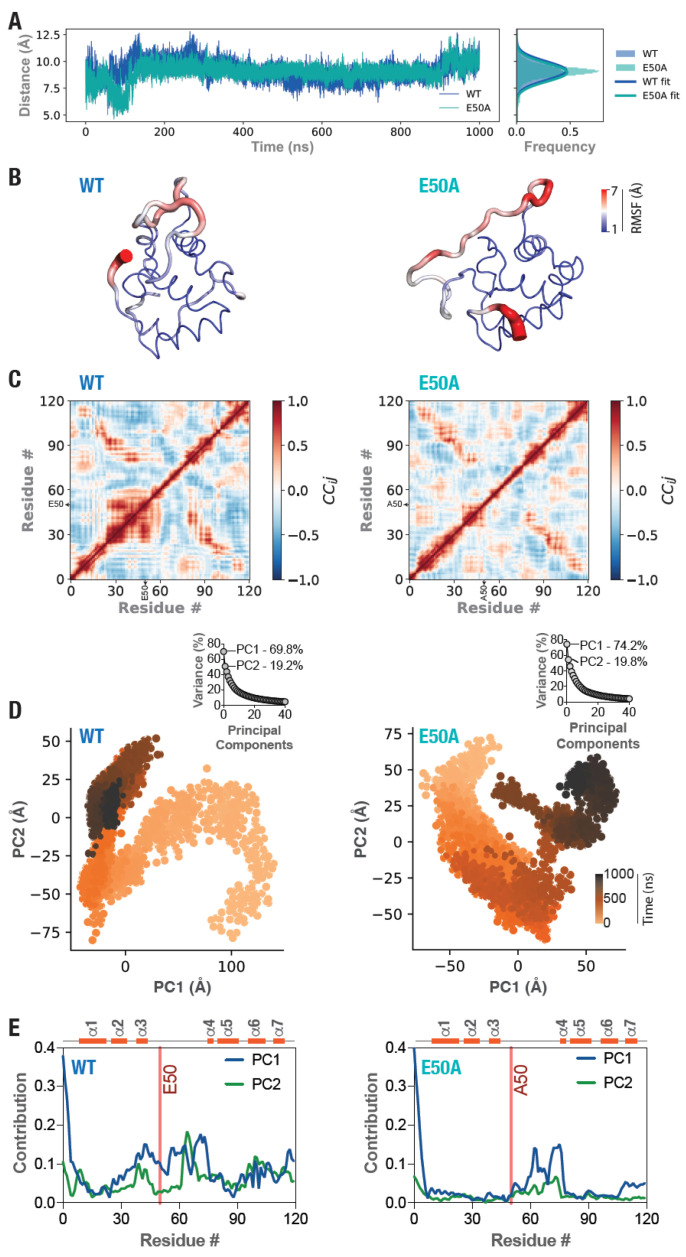
Altered structural dynamics in the E50A mutant picALuc. (**A**) Graphs showing Cα atom distances between positions 50 and 42 (E50-K42 in the WT and A50-K42 in the E50A mutant) of picALuc over a 1 μs GaMD simulation (left panel) and frequency distribution of the distances and their Gaussian fits (right panel). (**B**) Cartoon representation of the WT (left panel) and E50A mutant (right panel) picALuc with Cα atom RMSF values mapped on the conformers obtained after 1 μs of GaMD simulation (frame # 50,000) as b-factors. Color bar, Cα atom RMSF (Å). (**C**) Plots showing DCC of residues in the WT (left panel) and E50A mutant (right panel) picALuc determined from 1 μs GaMD trajectories of each protein. Location of the E50 and A50 residues are highlighted in the WT and the E50A mutant picALuc DCC graphs, respectively. (**D**) Graphs showing principal components 1 and 2 (PC1 and PC2) of the WT (left panel) and E50A mutant (right panel) determined from 1 μs GaMD trajectories of each protein. Color bar, time (ns). Insets: graphs showing percentage variance against principal components obtained from PCA analysis of the WT (left panel) and the E50A mutant (right panel) picALuc. Percentage variance explained by PC1 and PC2 are indicated in each case. (**E**) Graphs showing contribution of individual residues to the PC1 and PC2 in the WT (left panel) and the E50A mutant (right panel) picALuc simulations. Location of residue number 50 (E50 in the WT and A50 in the E50A mutant picALuc) is highlighted using a red line in each graph. Schematic above shows the location of various α-helices in the WT and the E50A mutant picALuc structural model used for the GaMD simulations.

The increased bioluminescence activity of the E50A mutant picALuc was intriguing and, therefore, we attempted to understand the underlying mechanism. For this, we introduced the E50A mutation in the structural model of picALuc and performed an all atom, explicit solvent 1 μs GaMD simulation similar to the WT protein (Figure S13; Supplementary Video S2). We then analyzed the trajectories of the WT and the E50A mutant picALuc to determine the distance between the Cα atoms in residues at position 50 (E in the WT and A in the E50A mutant picALuc) and 42 (K in both proteins) and found that the E50A mutation did not result in any major changes between the two proteins (9.1 ± 0.8 and 9.0 ± 0.8 Å for the WT and the E50A mutant picALuc, respectively, obtained from the Gaussian fitting of the frequency distributions of the distances; mean ± s.d.) except for some differences in the initial phases of the simulation (Figure 7A), suggesting that the salt bridge interaction between E50 and K42 residues frequently observed in the simulation trajectory of the WT picALuc is likely not playing any major role in the bioluminescence of the protein. We then analyzed the WT and the E50A mutant picALuc trajectory for Cα atom RMSF values and mapped the RMSF values of individual amino acid residues onto the structural conformer obtained from the last frame of the simulations of the respective protein as b-factors. This revealed an increased fluctuation in the N-terminal residues and the long loop between α-helices 3 and 4 in the E50A mutant picALuc compared to the WT picALuc (Figure 7B). We then performed DCC analysis of residues using the MD-TASK suite (version 1.0.1) of MD simulation analysis software [50] to elucidate any alterations in the correlated motions in the WT and E50A mutant picALuc proteins [32]. The WT picALuc showed both positively and negatively correlated motions in the residues with prominent correlations observed for residues close to the residue E50 (Figure 7C). However, such highly positively correlated motions were lost in the E50A mutant picALuc while some correlated motions were gained between the range of residues 36–49 and 6–17 (Figure 7C), suggesting a role for the residue E50 in the dynamics of the protein.

In order to further understand the structural dynamics of the WT and E50A mutant picALuc, we utilized the dimensionality reduction analysis and performed principal component analysis (PCA) of their trajectories, which is known to reveal collective motions in proteins [74] using the PCA module available in the Python (version 3.13.3)-based MDAnalysis package (version 2 or 2.9) [46,47]. Cumulative variance analysis of the principal components revealed the presence dominant collective motions in both the WT and the E50A mutant picALuc (Figure 7D) since principal components 1 and 2 (PC1 and PC2) could account for 69.8 and 19.2% of the variance in the WT protein while they could account for 74.2 and 19.8% of the variance in the E50A mutant protein. Importantly, this analysis revealed large differences in the PC1 and PC2 of the two trajectories (Figure 7C), suggesting changes in the collective dynamics of picALuc upon E50A mutation. We, therefore, determined the contribution of individual residues to the PC1 and PC2 in the WT and the E50A mutant picALuc trajectory and found residue E50, other than the N-terminal residues and those from the loop α3-α4, contributing significantly to PC1 in the WT protein (Figure 7D). Importantly, such contributions from A50, as well as those from α-helix 3 in general were lost in the PC1 of the E50A mutant picALuc (Figure 7D). These analyses were performed on the full 1 μs GaMD simulation trajectory. However, since the proteins showed large conformational changes in the initial phase of the simulation, we also performed analyses on the last 900 ns of the simulation trajectories to determine differences between the WT and E50A mutant picALuc after equilibration of their conformations in the simulation. This analysis also revealed large differences in the RMSF values as well as DCC between the WT and E50A mutant picALuc, as observed with the full 1 μs trajectory analysis (Figure S14A,B). Additionally, differences in the PC1 and PC2 were still observed between the WT and the E50A mutant picALuc and some contributions of the position 50 could be observed in the PC1 and PC2 of the WT but not the E50A mutant picALuc (Figure S14C,D). These data suggest a role for the residues E50 in the collective dynamics of picALuc, particularly in the initial phase of the simulation, and that this mode of collective motion likely restricts the catalytic activity of the WT picALuc while the altered collection motion in the E50A mutant picALuc likely reflects release of this restriction on the catalytic activity of the mutant protein.

### 3.6. Design of a picALuc-Based Split Protein for Monitoring PPI in Living Cells

**Figure 8:**
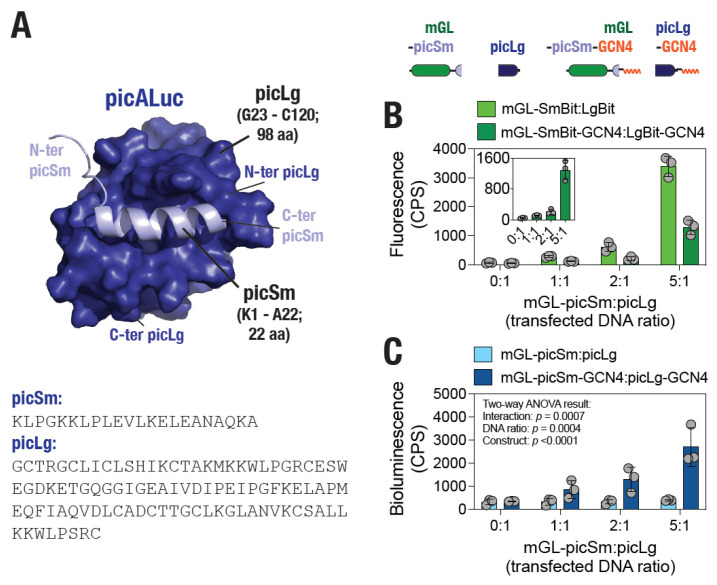
picALuc-based split protein for monitoring PPI. (**A**) Cartoon representation of a picALuc conformer showing the N-terminal, α-helical, small (picSm) and large (picLg) fragments generated for monitoring PPI. Bottom panel: amino acid residue sequences of the picSm and picLg fragments. (**B**) Top panel: schematic showing constructs generated for monitoring PPI (dimerization)-mediated bioluminescence activity. Bottom panel: graph showing fluorescence in cells transfected with the indicated ratios of picSM and picLg DNA. Inset: fluorescence in cells transfected with picSm-GCN4 and picLg-GCN4. Data shown are the mean ± s.d. of 3 measurements. (**C**) Graph showing bioluminescence activity in cells transfected with either mGL-picSm and picLg or mGL-picSm-GCN4 and picLg-GCN4 plasmid DNA at the indicated ratio (amount of plasmid DNA) showing increase in bioluminescence with increasing amount of the GCN4-fused picALuc fragments. Data shown are the mean ± s.d. from 3 independent experiments, with each experiment performed in triplicates. Two-way ANOVA *p* values show that changes in bioluminescence activity with increasing DNA ratios as well as between picSm:picLg and picSm-GCN4:picLg-GCN4 are significantly different.

Finally, we attempted to design a split protein to develop a protein fragment complementation-based assay platform for use in monitoring PPIs. For this, we analyzed the GaMD simulation trajectory of the WT picALuc to determine a stable conformation that can be utilized for designing the split protein. We utilized the *k*-means clustering algorithm to perform clustering (with *N* = 15, 20 or 25 clusters) of all conformers observed in the GaMD simulation trajectory. Each of the clustering revealed a significantly large number of conformers into one of the clusters (with 16, 18 and 22% of the conformers with *N* = 15, 20, and 25, respectively) with centroids around 600 ns of the simulation (Figures S15 and S16). We utilized the centroid of cluster obtained from clustering with *N* = 25 for further analysis and design of the split protein (Figure 2A). Specifically, we chose to utilize N-terminal residues spanning the α-helix 1 (Figure 1B) until residue A22 (one G residue N-terminal to the first disulfide bond forming C24 residue) (Figure S1C) as the smaller fragment named picSm (22 residue long; predicted molecular weight of 2.4 kDa) and the rest of the protein spanning residues G23 until C120 as the larger fragment named picLg (98 residue long; predicted molecular weight of 10.5 kDa) (Figure 8A). We note that the picSm fragment was not found to interact with the substrate docked on the picALuc conformation that was utilized for designing the split protein (Figure S17). We then designed a plasmid construct expressing the picSm fused with mGL at the N-terminal for monitoring expression levels of the protein and another expressing the picLg. Additionally, we generated a construct expressing mGL-picSm and picLg fused with the 19 residue long GCN4 leucine zipper peptide that is known to form a parallel, two-stranded coiled-coil structure [75,76] and has been utilized for synthetic dimerization applications including in BRET-based assays [77]. The latter two constructs were named mGL-picSm-GCN4 and picLg-GCN4, respectively. Transfection of HEK293T cells with increasing amounts of either mGL-picSm or mGL-picSm-GCN4 plasmid DNA, while maintaining the amount of picLg or picLg-GNC4, resulted in the increasing mGL fluorescence indicating increased expression of the picSm and picSm-GCN4 fragments (Figure 8B). Importantly, transfection of cells with increasing concentration of mGL-picSm-GCN4 resulted in increased bioluminescence, while such increases were not observed in cells transfected with mGL-picSm (Figure 8C). These results suggest that while picSm fragment likely do not possess high enough affinity for picLg to associate with it i.e. complement it and drive bioluminescent activity, GCN4-mediated dimerization likely brings the two fragments closer, enabling complementation and thus, bioluminescence activity. We note that while the picALuc SmBit is longer than the reported NLuc SmBit (22 vs. 11 residues), the picALuc LgBit is smaller than the NLuc LgBit (10.5 vs. 17.6 kDa) [78], and thus may serve as a better fusion tag for monitoring PPI, especially in the case proteins that are affected by fusion with larger protein tags.

## 4. Conclusions

To conclude, the structural modeling and MD simulation of picALuc, which was generated through the deletion of N- and C-terminal residues in ALuc [23], revealed a ‘compaction’ of the three-dimensional structure of the protein and provided insights into the possible residue-level interactions. Alanine mutation of selected residues that appeared to form salt bridge interaction revealed increased bioluminescence of the E50A mutant picALuc. Comparative analysis of the WT and E50A mutant picALuc GaMD simulation trajectories revealed an altered collective motion and specifically the contribution of position 50 in the mutant picALuc. Finally, clustering of the picALuc conformers and structural analysis allowed us to design a picALuc-based protein fragmentation assay that will find utility in monitoring PPI. We believe that the results presented here will pave the way for the generation of new variants of picALuc. This will certainly increase the applicability of the luciferase in a range of assays including gene expression, PPI, protein stability and BRET [1,2,3,4,5,6,79,80], as well as bioluminescence imaging that can be implemented in a number of biological and biomedical applications such as optogenetics [81,82,83,84] and drug screening [24,54,58]. We note that while similar MD simulation-based approaches may be utilized for understanding the structure–function relationship of luciferases, such approaches may not be generalizable to all luciferases due to differences in their sequence and structures. Broadly, the results presented here highlight the role of collective dynamics in picALuc activity and that mutations in the enzyme can be selected based on their contribution in the collective dynamics of the protein.

## Data Availability

The original contributions presented in this study are included in the article/Supplementary Materials. Further inquiries can be directed to the corresponding author.

## Supplementary Materials

The following supporting information can be downloaded at: https://www.mdpi.com/article/10.3390/bios16030167/s1, Table S1: H-bond occupancy table; Figure S1: Sequence alignments and disulfide bridges in picALuc; Figure S2: GLuc structural flexibility; Figure S3: Comparison of picALuc structural models generated using SWISS-MODEL and Alpha-Fold2. Figure S4: Secondary structure of picALuc from GaMD simulation; Figure S5: Stability of disulfide bridges in picALuc; Figure S6: Salt bridges observed in picALuc; Figure S7: Transient salt bridges observed in picALuc; Figure S8: Conventional MD simulation of picALuc; Figure S9: Salt bridges observed in conventional MD simulation of picALuc; Figure S10: GaMD simulation of ALuc; Figure S11: Bioluminescence characterization of mGL-picALuc constructs; Figure S12: SARS-CoV-2 M^pro^-mediated cleavage of mGL-M^pro^-Nter-picALuc biosensor; Figure S13: Structural dynamics of WT and E50A mutant picALuc; Figure S14: Collective dynamics of WT and E50A mutant picALuc obtained from the last 900 ns of GaMD simulations; Figure S15: Clustering analysis of picALuc conformers obtained from GaMD simulation trajectory; Figure S16: Conformational analysis of picALuc obtained from GaMD simulation; Figure S17: Docking of substrate on picALuc structural model; Video S1: picALuc-WT-1microsecond-GaMD-sim-traj; Video S2: picALuc-E50A-mutant-1microsecond-GaMD-sim-traj.
