# Supplementary material for: E50A Mutation Increases the Bioluminescence Activity of picALuc
Source: Biosensors (Basel). 2026 Mar 17;16(3):167. doi: 10.3390/bios16030167 (PMC13024344; doi:10.3390/bios16030167)
Supplement: Supplementary file 1 [file biosensors-16-00167-s001.zip › biosensors-4084323-supplementary.pdf]

biosensors
MDPI

Supporting Information

E50A Mutation Increases the Bioluminescence Activity of pi-cALuc

Kabir H. Biswas[1,*]

Affiliation:
[1]College of Health & Life Sciences, Hamad Bin Khalifa University, Doha, Qatar

ORCID:
Kabir H. Biswas: 0000-0001-9194-4127

***Correspondence:** kbiswas@hbku.edu.qa

Supplementary Text

GaMD simulation protocol

```
cutoff 12.0
pairlistdist 14.0
switching on
switchdist 10.0
PME on
PMEGridspacing 1
wrapAll on
wrapWater on
############################################################################
#cr
#cr            (C) Copyright 1995-2009 The Board of Trustees of the
#cr                        University of Illinois
#cr                         All Rights Reserved
#cr
############################################################################

############################################################################
# RCS INFORMATION:
#
#       $RCSfile: MD.conf,v $
#       $Author: jribeiro $        $Locker:    $             $State: Exp $
#       $Revision: 1.2 $       $Date: 2017/05/10 19:03:08 $
#
############################################################################
##START HERE###
##Simulation Template##
# Simulation conditions
coordinates 7D2O-picALuc-minbox-37deg_QwikMD.pdb
structure 7D2O-picALuc-minbox-37deg_QwikMD.psf

binCoordinates Equilibration.restart.coor
binVelocities Equilibration.restart.vel
extendedSystem Equilibration.restart.xsc

# Simulation conditions
#temperature 0

# Harmonic constraints
constraints off
```

```
consref qwikmdTemp_constraints.pdb
conskfile qwikmdTemp_constraints.pdb
constraintScaling 2
consexp 2
conskcol B


# Output Parameters
binaryoutput no
outputname MD
outputenergies 400
outputtiming 400
outputpressure 400
binaryrestart yes
dcdfile MD.dcd
dcdfreq 10000
XSTFreq 10000
restartfreq 10000
restartname MD.restart


# Thermostat Parameters
langevin on
langevintemp 310
langevinHydrogen        off
langevindamping 1

# Barostat Parameters
usegrouppressure yes
useflexiblecell no
useConstantArea no
langevinpiston on
langevinpistontarget 1.01325
langevinpistonperiod 200
langevinpistondecay 100
langevinpistontemp 310

# Integrator Parameters
timestep 2
firstTimestep 0
fullElectFrequency 2
nonbondedfreq 1
stepspercycle 10
```

https://doi.org/10.3390/bios16030167

```
# Force Field Parameters
paratypecharmm on
parameters toppar_water_ions_namd.str
parameters toppar_all36_carb_glycopeptide.str
parameters par_all36_lipid.prm
parameters par_all36_na.prm
parameters par_all36_prot.prm
parameters par_all36_carb.prm
parameters par_all36_cgenff.prm
exclude scaled1-4
1-4scaling 1.0
rigidbonds all


#Implicit Solvent Parameters
gbis off
alphaCutoff           14.0
ionConcentration      0.15

accelMD               on
accelMDdual           on
accelMDdihe           on
accelMDG              on
accelMDGiE            1
accelMDGRestart       off
accelMDGcMDSteps      1000000
accelMDGEquiSteps     25000000
accelMDGcMDPrepSteps        200000
accelMDGEquiPrepSteps       200000
accelMDOutFreq    10000
accelMDGsigma0P 6.0
accelMDGsigma0D 6.0
accelMDGrestartfile         GaMD.restart.gamd

# Script
run 500000000
set file [open MD.check w+]
set done 1
if {[file exists MD.restart.coor] != 1 || [file exists MD.restart.vel] != 1 || [file exists MD.restart.xsc] != 1 } {
        set done 0
}
if {$done == 1} {
        puts $file "DONE"
    flush $file
```

```
    close $file
} else {
            puts $file "One or more files failed to be written"
      flush $file
    close $file
}
```

Nucleotide and amino acid sequences:

mGL-picALuc nucleotide sequence:

ATGGGAAGTTCACATCATCATCATCATCACTCATCAGGACTGGTGCCAC-
GGGGGTCTGAATTCGGCATGGTGAGCAAGGGCGAGGAGCTGTTCACCGGGGTGGTGCCCATCCTGGTCGAGCTGGACGGCGACGTAAAC
GGCCACAAGTTCAGCGTCCGCGGCGAGGGCGAGGGCGATGCCACCAACGGCAA-
GCTGACCCTGAAGTTCATCTGCACCACCGGCAAGCTGCCCGTGCCCTGGCCCACCCTCGTGACCACCTTAGGCTACGGCGTGGCCTGCTTC
GCCCGCTACCCCGACCACATGAAGCAGCACGACTTCTTCAAGTCCGCCATGCCCGAAGGC-
TACGTCCAGGAGCGCACCATCTCTTTCAAGGACGACGGTACCTACAAGACCCGCGCCGAGGTGAAGTTCGAGGGCGACACCCTGGTGAAC
CGCATCGTGCTGAAGGGCATCGACTTCAAGGAGGACGGCAACATCCTGGGGCACAAGCTG-
GAGTACAACTTCAACAGCCACAAGGTCTATATCACGGCCGACAAGCAGAAGAACGGCATCAAGGCTAACTTCAAGACCCGCCACAACGT
TGAGGACGGCGGCGTGCAGCTCGCCGACCACTACCAGCAGAACACCCCCATCGGCGAC-
GGCCCCGTGCTGCTGCCCGACAACCACTACCTGAGCCATCAGTCCAAACTGAGCAAAGACCCCAACGAGAAGCGCGATCACATGGTCCT
GAAGGAGAGGGTGACCGCCGCCGGGATTACACATGACATGGACGAGCTGTACAAGTAC-
GGATCCGCGGCCGCCACCGAGAACCTGTATGCAGTGCTCCAAAGCGGATTTCGCGGCTCTGGCAGCGCTATGAAGCTGCCCGGCAAGAAG
CTGCCCCTGGAGGTGCTGAAGGAGCTGGAGGCCAACGCCCAGAAGGCCGGCTGCAC-
CAGGGGCTGCCTGATCTGCCTGAGCCACATCAAGTGCACCGCCAAGATGAAGAAGTGGCTGCCCGGCAGGTGCGAGAGCTGGGAGGGCG
ACAAGGAGACCGGCCAGGGCGGCATCGGCGAGGCCATCGTGGACATCCCCGA-
GATCCCCGGCTTCAAGGAGCTGGCCCCCATGGAGCAGTTCATCGCCCAGGTGGACCTGTGCGCCGACTGCACCACCGGCTGCCTGAAGGG
CCTGGCCAACGTGAAGTGCAGCGCCCTGCTGAAGAAGTGGCTGCCCAGCAGGTGCGGTAC-
CGACTACAAAGACCATGACGGTGATTATAAAGATCATGACATCGATTACAAGGATGACGATGACAAGGATATCTGA

mGL-picALuc amino acid sequence:

MGSSHHHHHHSSGLVPRGSEFGMVSKGEELFTGVVPILVELDGDVNGHKFSVRGEGEG-
DATNGKLTLKFICTTGKLPVPWPTLVTTLGYGVACFARYPDHMKQHDFFKSAMPEGYVQERTISFKDDGTYKTRAEVKFEGDTLVNRIVLKGIDFK
EDGNILGHKLEYNFNSHKVYITADKQKNGIKANFKTRHN-
VEDGGVQLADHYQQNTPIGDGPVLLPDNHYLSHQSKLSKDPNEKRDHMVLKERVTAAGITHDMDELYKYGSAAATENLYAVLQSGFRGSGSAM
KLPGKKLPLEVLKELEANAQKAGCTRGCLICLSHIKCTAKMKKWLPGRCESWEGDKETGQG-
GIGEAIVDIPEIPGFKELAPMEQFIAQVDLCADCTTGCLKGLANVKCSALLKKWLPSRCGTDYKDHDGDYKDHDIDYKDDDDKDI*

mGL-picALuc(E10A) nucleotide sequence:

ATGGGAAGTTCACATCATCATCATCATCACTCATCAGGACTGGTGCCACGGGGGTCTGAATTCGGCATGGTGAGCAAGGGCGAGGAGCTG
TTCACCGGGGTGGTGCCCATCCTGGTCGAGCTGGACGGCGACGTAAACGGCCACAAGTTCAGCGTCCGCGGCGAGGGCGAGGGCGATGC
CACCAACGGCAAGCTGACCCTGAAGTTCATCTGCACCACCGGCAAGCTGCCCGTGCCCTGGCCCACCCTCGTGACCACCTTAGGCTACGG
CGTGGCCTGCTTCGCCCGCTACCCCGACCACATGAAGCAGCACGACTTCTTCAAGTCCGCCATGCCCGAAGGCTACGTCCAGGAGCGCAC
CATCTCTTTCAAGGACGACGGTACCTACAAGACCCGCGCCGAGGTGAAGTTCGAGGGCGACACCCTGGTGAACCGCATCGTGCTGAAGGG
CATCGACTTCAAGGAGGACGGCAACATCCTGGGGCACAAGCTGGAGTACAACTTCAACAGCCACAAGGTCTATATCACGGCCGACAAGC
AGAAGAACGGCATCAAGGCTAACTTCAAGACCCGCCACAACGTTGAGGACGGCGGCGTGCAGCTCGCCGACCACTACCAGCAGAACACC
CCCATCGGCGACGGCCCCGTGCTGCTGCCCGACAACCACTACCTGAGCCATCAGTCCAAACTGAGCAAAGACCCCAACGAGAAGCGCGA
TCACATGGTCCTGAAGGAGAGGGTGACCGCCGCCGGGATTACACATGACATGGACGAGCTGTACAAGTACGGATCCGCGGCCGCCACCG
AGAACCTGTATGCAGTGCTCCAAAGCGGATTTCGCGGCTCTGGCAGCGCTATGAAGCTGCCCGGCAAGAAGCTGCCCCTGGCCGTGCTGA
AGGAGCTGGAGGCCAACGCCCAGAAGGCCGGCTGCACCAGGGGCTGCCTGATCTGCCTGAGCCACATCAAGTGCACCGCCAAGATGAAG
AAGTGGCTGCCCGGCAGGTGCGAGAGCTGGGAGGGCGACAAGGAGACCGGCCAGGGCGGCATCGGCGAGGCCATCGTGGACATCCCCG
AGATCCCCGGCTTCAAGGAGCTGGCCCCCATGGAGCAGTTCATCGCCCAGGTGGACCTGTGCGCCGACTGCACCACCGGCTGCCTGAAGG
GCCTGGCCAACGTGAAGTGCAGCGCCCTGCTGAAGAAGTGGCTGCCCAGCAGGTGCGGTACCGACTACAAAGACCATGACGGTGATTAT
AAAGATCATGACATCGATTACAAGGATGACGATGACAAGGATATCTGA

mGL-picALuc(E10A) amino acid sequence:

MGSSHHHHHHSSGLVPRGSEFGMVSKGEELFTGVVPILVELDGDVNGHKFSVRGEGEGDATNGKLTLKFICTTGKLPVPWPTLVTTLGYGVACFA
RYPDHMKQHDFFKSAMPEGYVQERTISFKDDGTYKTRAEVKFEGDTLVNRIVLKGIDFKEDGNILGHKLEYNFNSHKVYITADKQKNGIKANFKT
RHNVEDGGVQLADHYQQNTPIGDGPVLLPDNHYLSHQSKLSKDPNEKRDHMVLKERVTAAGITHDMDELYKYGSAAATENLYAVLQSGFRGSG
SAMKLPGKKLPLAVLKELEANAQKAGCTRGCLICLSHIKCTAKMKKWLPGRCESWEGDKETGQGGIGEAIVDIPEIPGFKELAPMEQFIAQVDLCA
DCTTGCLKGLANVKCSALLKKWLPSRCGTDYKDHDGDYKDHDIDYKDDDDKDI*

mGL-picALuc(E50A) nucleotide sequence:

ATGGGAAGTTCACATCATCATCATCATCACTCATCAGGACTGGTGCCACGGGGGTCTGAATTCGGCATGGTGAGCAAGGGCGAGGAGCTG
TTCACCGGGGTGGTGCCCATCCTGGTCGAGCTGGACGGCGACGTAAACGGCCACAAGTTCAGCGTCCGCGGCGAGGGCGAGGGCGATGC
CACCAACGGCAAGCTGACCCTGAAGTTCATCTGCACCACCGGCAAGCTGCCCGTGCCCTGGCCCACCCTCGTGACCACCTTAGGCTACGG
CGTGGCCTGCTTCGCCCGCTACCCCGACCACATGAAGCAGCACGACTTCTTCAAGTCCGCCATGCCCGAAGGCTACGTCCAGGAGCGCAC
CATCTCTTTCAAGGACGACGGTACCTACAAGACCCGCGCCGAGGTGAAGTTCGAGGGCGACACCCTGGTGAACCGCATCGTGCTGAAGGG
CATCGACTTCAAGGAGGACGGCAACATCCTGGGGCACAAGCTGGAGTACAACTTCAACAGCCACAAGGTCTATATCACGGCCGACAAGC
AGAAGAACGGCATCAAGGCTAACTTCAAGACCCGCCACAACGTTGAGGACGGCGGCGTGCAGCTCGCCGACCACTACCAGCAGAACACC
CCCATCGGCGACGGCCCCGTGCTGCTGCCCGACAACCACTACCTGAGCCATCAGTCCAAACTGAGCAAAGACCCCAACGAGAAGCGCGA
TCACATGGTCCTGAAGGAGAGGGTGACCGCCGCCGGGATTACACATGACATGGACGAGCTGTACAAGTACGGATCCGCGGCCGCCACCG
AGAACCTGTATGCAGTGCTCCAAAGCGGATTTCGCGGCTCTGGCAGCGCTATGAAGCTGCCCGGCAAGAAGCTGCCCCTGGAGGTGCTGA
AGGAGCTGGAGGCCAACGCCCAGAAGGCCGGCTGCACCAGGGGCTGCCTGATCTGCCTGAGCCACATCAAGTGCACCGCCAAGATGAAG
AAGTGGCTGCCCGGCAGGTGCGCCAGCTGGGAGGGCGACAAGGAGACCGGCCAGGGCGGCATCGGCGAGGCCATCGTGGACATCCCCG
AGATCCCCGGCTTCAAGGAGCTGGCCCCCATGGAGCAGTTCATCGCCCAGGTGGACCTGTGCGCCGACTGCACCACCGGCTGCCTGAAGG
GCCTGGCCAACGTGAAGTGCAGCGCCCTGCTGAAGAAGTGGCTGCCCAGCAGGTGCGGTACCGACTACAAAGACCATGACGGTGATTAT
AAAGATCATGACATCGATTACAAGGATGACGATGACAAGGATATCTGA

mGL-picALuc(E50A) amino acid sequence:

MGSSHHHHHHSSGLVPRGSEFGMVSKGEELFTGVVPILVELDGDVNGHKFSVRGEGEGDATNGKLTLKFICTTGKLPVPWPTLVTTLGYGVACFA
RYPDHMKQHDFFKSAMPEGYVQERTISFKDDGTYKTRAEVKFEGDTLVNRIVLKGIDFKEDGNILGHKLEYNFNSHKVYITADKQKNGIKANFKT
RHNVEDGGVQLADHYQQNTPIGDGPVLLPDNHYLSHQSKLSKDPNEKRDHMVLKERVTAAGITHDMDELYKYGSAAATENLYAVLQSGFRGSG
SAMKLPGKKLPLEVLKELEANAQKAGCTRGCLICLSHIKCTAKMKKWLPGRCASWEGDKETGQGGIGEAIVDIPEIPGFKELAPMEQFIAQVDLCA
DCTTGCLKGLANVKCSALLKKWLPSRCGTDYKDHDGDYKDHDIDYKDDDDKDI*

mGL-picALuc(D94A) nucleotide sequence:

ATGGGAAGTTCACATCATCATCATCATCACTCATCAGGACTGGTGCCACGGGGGTCTGAATTCGGCATGGTGAGCAAGGGCGAGGAGCTG
TTCACCGGGGTGGTGCCCATCCTGGTCGAGCTGGACGGCGACGTAAACGGCCACAAGTTCAGCGTCCGCGGCGAGGGCGAGGGCGATGC
CACCAACGGCAAGCTGACCCTGAAGTTCATCTGCACCACCGGCAAGCTGCCCGTGCCCTGGCCCACCCTCGTGACCACCTTAGGCTACGG
CGTGGCCTGCTTCGCCCGCTACCCCGACCACATGAAGCAGCACGACTTCTTCAAGTCCGCCATGCCCGAAGGCTACGTCCAGGAGCGCAC
CATCTCTTTCAAGGACGACGGTACCTACAAGACCCGCGCCGAGGTGAAGTTCGAGGGCGACACCCTGGTGAACCGCATCGTGCTGAAGGG
CATCGACTTCAAGGAGGACGGCAACATCCTGGGGCACAAGCTGGAGTACAACTTCAACAGCCACAAGGTCTATATCACGGCCGACAAGC
AGAAGAACGGCATCAAGGCTAACTTCAAGACCCGCCACAACGTTGAGGACGGCGGCGTGCAGCTCGCCGACCACTACCAGCAGAACACC
CCCATCGGCGACGGCCCCGTGCTGCTGCCCGACAACCACTACCTGAGCCATCAGTCCAAACTGAGCAAAGACCCCAACGAGAAGCGCGA
TCACATGGTCCTGAAGGAGAGGGTGACCGCCGCCGGGATTACACATGACATGGACGAGCTGTACAAGTACGGATCCGCGGCCGCCACCG
AGAACCTGTATGCAGTGCTCCAAAGCGGATTTCGCGGCTCTGGCAGCGCTATGAAGCTGCCCGGCAAGAAGCTGCCCCTGGAGGTGCTGA
AGGAGCTGGAGGCCAACGCCCAGAAGGCCGGCTGCACCAGGGGCTGCCTGATCTGCCTGAGCCACATCAAGTGCACCGCCAAGATGAAG
AAGTGGCTGCCCGGCAGGTGCGAGAGCTGGGAGGGCGACAAGGAGACCGGCCAGGGCGGCATCGGCGAGGCCATCGTGGACATCCCCG
AGATCCCCGGCTTCAAGGAGCTGGCCCCCATGGAGCAGTTCATCGCCCAGGTGGACCTGTGCGCCGCCTGCACCACCGGCTGCCTGAAGG
GCCTGGCCAACGTGAAGTGCAGCGCCCTGCTGAAGAAGTGGCTGCCCAGCAGGTGCGGTACCGACTACAAAGACCATGACGGTGATTAT
AAAGATCATGACATCGATTACAAGGATGACGATGACAAGGATATCTGA

mGL-picALuc(D94A) amino acid sequence:

MGSSHHHHHHSSGLVPRGSEFGMVSKGEELFTGVVPILVELDGDVNGHKFSVRGEGEGDATNGKLTLKFICTTGKLPVPWPTLVTTLGYGVACFA
RYPDHMKQHDFFKSAMPEGYVQERTISFKDDGTYKTRAEVKFEGDTLVNRIVLKGIDFKEDGNILGHKLEYNFNSHKVYITADKQKNGIKANFKT
RHNVEDGGVQLADHYQQNTPIGDGPVLLPDNHYLSHQSKLSKDPNEKRDHMVLKERVTAAGITHDMDELYKYGSAAATENLYAVLQSGFRGSG
SAMKLPGKKLPLEVLKELEANAQKAGCTRGCLICLSHIKCTAKMKKWLPGRCESWEGDKETGQGGIGEAIVDIPEIPGFKELAPMEQFIAQVDLCA
ACTTGCLKGLANVKCSALLKKWLPSRCGTDYKDHDGDYKDHDIDYKDDDDKDI*

mGL-picSm nucleotide sequence:

ATGGGAAGTTCACATCATCATCATCATCACTCATCAGGACTGGTGCCACGGGGGTCTGAATTCGGCATGGTGAGCAAGGGCGAGGAGCTG
TTCACCGGGGTGGTGCCCATCCTGGTCGAGCTGGACGGCGACGTAAACGGCCACAAGTTCAGCGTCCGCGGCGAGGGCGAGGGCGATGC
CACCAACGGCAAGCTGACCCTGAAGTTCATCTGCACCACCGGCAAGCTGCCCGTGCCCTGGCCCACCCTCGTGACCACCTTAGGCTACGG
CGTGGCCTGCTTCGCCCGCTACCCCGACCACATGAAGCAGCACGACTTCTTCAAGTCCGCCATGCCCGAAGGCTACGTCCAGGAGCGCAC
CATCTCTTTCAAGGACGACGGTACCTACAAGACCCGCGCCGAGGTGAAGTTCGAGGGCGACACCCTGGTGAACCGCATCGTGCTGAAGGG
CATCGACTTCAAGGAGGACGGCAACATCCTGGGGCACAAGCTGGAGTACAACTTCAACAGCCACAAGGTCTATATCACGGCCGACAAGC
AGAAGAACGGCATCAAGGCTAACTTCAAGACCCGCCACAACGTTGAGGACGGCGGCGTGCAGCTCGCCGACCACTACCAGCAGAACACC
CCCATCGGCGACGGCCCCGTGCTGCTGCCCGACAACCACTACCTGAGCCATCAGTCCAAACTGAGCAAAGACCCCAACGAGAAGCGCGA
TCACATGGTCCTGAAGGAGAGGGTGACCGCCGCCGGGATTACACATGACATGGACGAGCTGTACAAGTACGGATCCGCGGCCGCCACCG

AGAACCTGTATGCAGTGCTCCAAAGCGGATTTCGCGGCTCTGGCAGCGCTATGAAGCTGCCCGGCAAGAAGCTGCCCCTGGAGGTGCTGA
AGGAGCTGGAGGCCAACGCCCAGAAGGCCTGA

mGL-picSm amino acid sequence:

MGSSHHHHHHSSGLVPRGSEFGMVSKGEELFTGVVPILVELDGDVNGHKFSVRGEGEGDATNGKLTLKFICTTGKLPVPWPTLVTTLGYGVACFA
RYPDHMKQHDFFKSAMPEGYVQERTISFKDDGTYKTRAEVKFEGDTLVNRIVLKGIDFKEDGNILGHKLEYNFNSHKVYITADKQKNGIKANFKT
RHNVEDGGVQLADHYQQNTPIGDGPVLLPDNHYLSHQSKLSKDPNEKRDHMVLKERVTAAGITHDMDELYKYGSAAATENLYAVLQSGFRGSG
SAMKLPGKKLPLEVLKELEANAQKA*

mGL-picSm-GNC4 nucleotide sequence:

ATGGGAAGTTCACATCATCATCATCATCACTCATCAGGACTGGTGCCACGGGGGTCTGAATTCGGCATGGTGAGCAAGGGCGAGGAGCTG
TTCACCGGGGTGGTGCCCATCCTGGTCGAGCTGGACGGCGACGTAAACGGCCACAAGTTCAGCGTCCGCGGCGAGGGCGAGGGCGATGC
CACCAACGGCAAGCTGACCCTGAAGTTCATCTGCACCACCGGCAAGCTGCCCGTGCCCTGGCCCACCCTCGTGACCACCTTAGGCTACGG
CGTGGCCTGCTTCGCCCGCTACCCCGACCACATGAAGCAGCACGACTTCTTCAAGTCCGCCATGCCCGAAGGCTACGTCCAGGAGCGCAC
CATCTCTTTCAAGGACGACGGTACCTACAAGACCCGCGCCGAGGTGAAGTTCGAGGGCGACACCCTGGTGAACCGCATCGTGCTGAAGGG
CATCGACTTCAAGGAGGACGGCAACATCCTGGGGCACAAGCTGGAGTACAACTTCAACAGCCACAAGGTCTATATCACGGCCGACAAGC
AGAAGAACGGCATCAAGGCTAACTTCAAGACCCGCCACAACGTTGAGGACGGCGGCGTGCAGCTCGCCGACCACTACCAGCAGAACACC
CCCATCGGCGACGGCCCCGTGCTGCTGCCCGACAACCACTACCTGAGCCATCAGTCCAAACTGAGCAAAGACCCCAACGAGAAGCGCGA
TCACATGGTCCTGAAGGAGAGGGTGACCGCCGCCGGGATTACACATGACATGGACGAGCTGTACAAGTACGGATCCGCGGCCGCCACCG
AGAACCTGTATGCAGTGCTCCAAAGCGGATTTCGCGGCTCTGGCAGCGCTATGAAGCTGCCCGGCAAGAAGCTGCCCCTGGAGGTGCTGA
AGGAGCTGGAGGCCAACGCCCAGAAGGCCGGCTCTGGCTCTATCGATGGCTCTGGCTCTGAAGAACTGCTGAGCAAAAACTATCATCTGG
AAAACGAAGTGGCGCGCCTGAAAAAACTGGTGGGCGAACGCTGA

mGL-picSm-GCN4 amino acid sequence:

MGSSHHHHHHSSGLVPRGSEFGMVSKGEELFTGVVPILVELDGDVNGHKFSVRGEGEGDATNGKLTLKFICTTGKLPVPWPTLVTTLGYGVACFA
RYPDHMKQHDFFKSAMPEGYVQERTISFKDDGTYKTRAEVKFEGDTLVNRIVLKGIDFKEDGNILGHKLEYNFNSHKVYITADKQKNGIKANFKT
RHNVEDGGVQLADHYQQNTPIGDGPVLLPDNHYLSHQSKLSKDPNEKRDHMVLKERVTAAGITHDMDELYKYGSAAATENLYAVLQSGFRGSG
SAMKLPGKKLPLEVLKELEANAQKAGSGSIDGSGSEELLSKNYHLENEVARLKKLVGER*

picLg nucleotide sequence:

ATGGGCTGCACCAGGGGCTGCCTGATCTGCCTGAGCCACATCAAGTGCACCGCCAAGATGAAGAAGTGGCTGCCCGGCAGGTGCGAGAG
CTGGGAGGGCGACAAGGAGACCGGCCAGGGCGGCATCGGCGAGGCCATCGTGGACATCCCCGAGATCCCCGGCTTCAAGGAGCTGGCCC
CCATGGAGCAGTTCATCGCCCAGGTGGACCTGTGCGCCGACTGCACCACCGGCTGCCTGAAGGGCCTGGCCAACGTGAAGTGCAGCGCCC
TGCTGAAGAAGTGGCTGCCCAGCAGGTGCGGTACCGACTACAAAGACCATGACGGTGATTATAAAGATCATGACATCGATTACAAGGAT
GACGATGACAAGGGATCCTTAAGGATATCTGAGCGGCCGCGAATTCCTCGAGTCTAG

picLg amino acid sequence:

MGCTRGCLICLSHIKCTAKMKKWLPGRCESWEGDKETGQGGIGEAIVDIPEIPGFKELAPMEQFIAQVDLCADCTTGCLKGLANVKCSALLKKWL
PSRCGTDYKDHDGDYKDHDIDYKDDDDKGSLRISERPRIPRV*

picLg-GNC4 nucleotide sequence:

ATGGGCTGCACCAGGGGCTGCCTGATCTGCCTGAGCCACATCAAGTGCACCGCCAAGATGAAGAAGTGGCTGCCCGGCAGGTGCGAGAG
CTGGGAGGGCGACAAGGAGACCGGCCAGGGCGGCATCGGCGAGGCCATCGTGGACATCCCCGAGATCCCCGGCTTCAAGGAGCTGGCCC

https://doi.org/10.3390/bios16030167

CCATGGAGCAGTTCATCGCCCAGGTGGACCTGTGCGCCGACTGCACCACCGGCTGCCTGAAGGGCCTGGCCAACGTGAAGTGCAGCGCCC
TGCTGAAGAAGTGGCTGCCCAGCAGGTGCGGTACCGGCTCTGGCTCTGGCTCTGGCTCTGGCTCTGAAGAACTGCTGAGCAAAAACTATC
ATCTGGAAAACGAAGTGGCGCGCCTGAAAAAACTGGTGGGCGAACGCGACTACAAAGACCATGACGGTGATTATAAAGATCATGACATC
GATTACAAGGATGACGATGACAAGGGATCCTTAAGGATATCTGAGCGGCCGCGAATTCCTCGAGTCTAG

picLg-GCN4 amino acid sequence:

MGCTRGCLICLSHIKCTAKMKKWLPGRCESWEGDKETGQGGIGEAIVDIPEIPGFKELAPMEQFIAQVDLCADCTTGCLKGLANVKCSALLKKWL
PSRCGTGSGSGSGSGSEELLSKNYHLENEVARLKKLVGERDYKDHDGDYKDHDIDYKDDDDKGSLRISERPRIPRV*

Supplementary Table

Table S1. H-bond occupancy table. H-bonds showing >5% occupancy. H-bonds formed Glu50 side chain are highlighted in green while that formed by its main chain is highlighted in light green. Similarly, H-bonds formed by Glu10 side chain are highlighted in blue while that formed by its main chain is highlighted in light blue. H-bonds formed by Asp94 side chain is highlighted in grey.

#	Donor	Acceptor	Occupancy
1	LYS36-Side	ASP55-Side	91.31%
2	ARG119-Side	GLU53-Side	75.32%
3	LYS42-Side	GLU50-Side	68.80%
4	GLU16-Main	LEU12-Main	65.42%
5	ASN18-Main	GLU14-Main	62.57%
6	LEU12-Main	PRO8-Main	60.13%
7	LYS13-Main	LEU9-Main	57.13%
8	LEU15-Main	VAL11-Main	55.16%
9	ARG26-Side	GLU16-Side	52.80%
10	LYS101-Main	ALA22-Main	51.20%
11	ALA19-Main	LEU15-Main	48.66%
12	SER109-Side	GLU14-Side	44.72%
13	LEU45-Main	MET41-Main	44.70%
14	LEU32-Main	CYS28-Main	44.50%
15	SER33-Side	LEU29-Main	41.72%
16	CYS24-Main	ALA19-Main	40.07%
17	TRP52-Side	LYS5-Main	38.88%
18	ALA22-Main	ASN18-Main	37.40%
19	GLN20-Main	GLU16-Main	36.60%
20	LYS42-Main	THR38-Main	35.86%
21	LYS113-Side	CYS120-Side	33.52%
22	ALA17-Main	LYS13-Main	33.06%
23	LYS21-Main	ALA17-Main	33.02%
24	ASP55-Main	LEU2-Main	32.54%
25	ILE30-Main	ARG26-Main	31.20%
26	CYS31-Main	GLY27-Main	29.20%
27	LYS1-Main	ASP55-Side	27.20%
28	LYS56-Side	ASP94-Side	23.18%
29	GLN60-Side	ASP55-Side	21.64%
30	LYS43-Main	ALA39-Main	18.49%
31	LYS6-Side	GLU57-Side	18.48%
32	ALA93-Main	GLY61-Main	18.44%
41	ALA110-Main	GLU14-Side	11.52%
42	LYS13-Side	GLU10-Side	11.45%
43	LYS56-Side	THR58-Side	11.33%
44	GLN84-Side	PHE76-Main	11.12%
45	ARG48-Side	CYS120-Side	11.00%
46	HSD34-Side	GLY54-Main	11.00%
47	GLY59-Main	LYS56-Main	10.59%
48	LYS36-Side	GLY54-Main	10.06%
49	LYS56-Side	GLU57-Side	9.78%
50	LYS13-Side	GLU16-Side	9.65%
51	THR97-Side	THR96-Main	9.55%
52	LEU103-Main	CYS99-Main	8.63%
53	LYS77-Side	GLU72-Side	8.31%
54	LEU29-Main	THR25-Main	8.06%
55	LYS56-Main	GLN60-Side	7.81%
56	ASN18-Side	GLU14-Main	7.33%
57	LYS113-Main	SER109-Main	7.08%
58	THR96-Side	ALA93-Main	7.00%
59	CYS37-Main	GLU50-Side	6.72%
60	LYS36-Side	ASP69-Side	6.72%
61	ARG48-Side	VAL106-Main	6.68%
62	LYS40-Side	LEU79-Main	6.60%
63	LYS114-Main	ALA110-Main	6.53%
64	LEU116-Main	LEU112-Main	6.35%
65	CYS95-Main	CYS92-Main	6.13%
66	GLY4-Main	ASP55-Main	5.96%
67	THR38-Side	GLN84-Side	5.75%
68	TRP52-Main	SER33-Main	5.72%
69	LYS36-Side	GLU50-Side	5.70%
70	LYS5-Side	GLU53-Side	5.60%
71	LYS107-Main	ASN18-Side	5.51%
72	ASP55-Main	LYS1-Main	5.47%

33	GLU50-Main	ILE35-Main	17.86%
34	LEU100-Main	GLY23-Main	15.76%
35	GLU14-Main	GLU10-Main	15.16%
36	GLN88-Side	LYS36-Main	15.04%
37	TRP115-Main	LEU111-Main	13.46%
38	SER33-Main	LEU29-Main	12.73%
39	ARG48-Side	PRO46-Main	12.18%
40	TRP44-Main	LYS40-Main	11.90%
73	LEU111-Main	GLU14-Side	5.40%
74	MET41-Main	THR38-Side	5.19%

Supplementary Figures

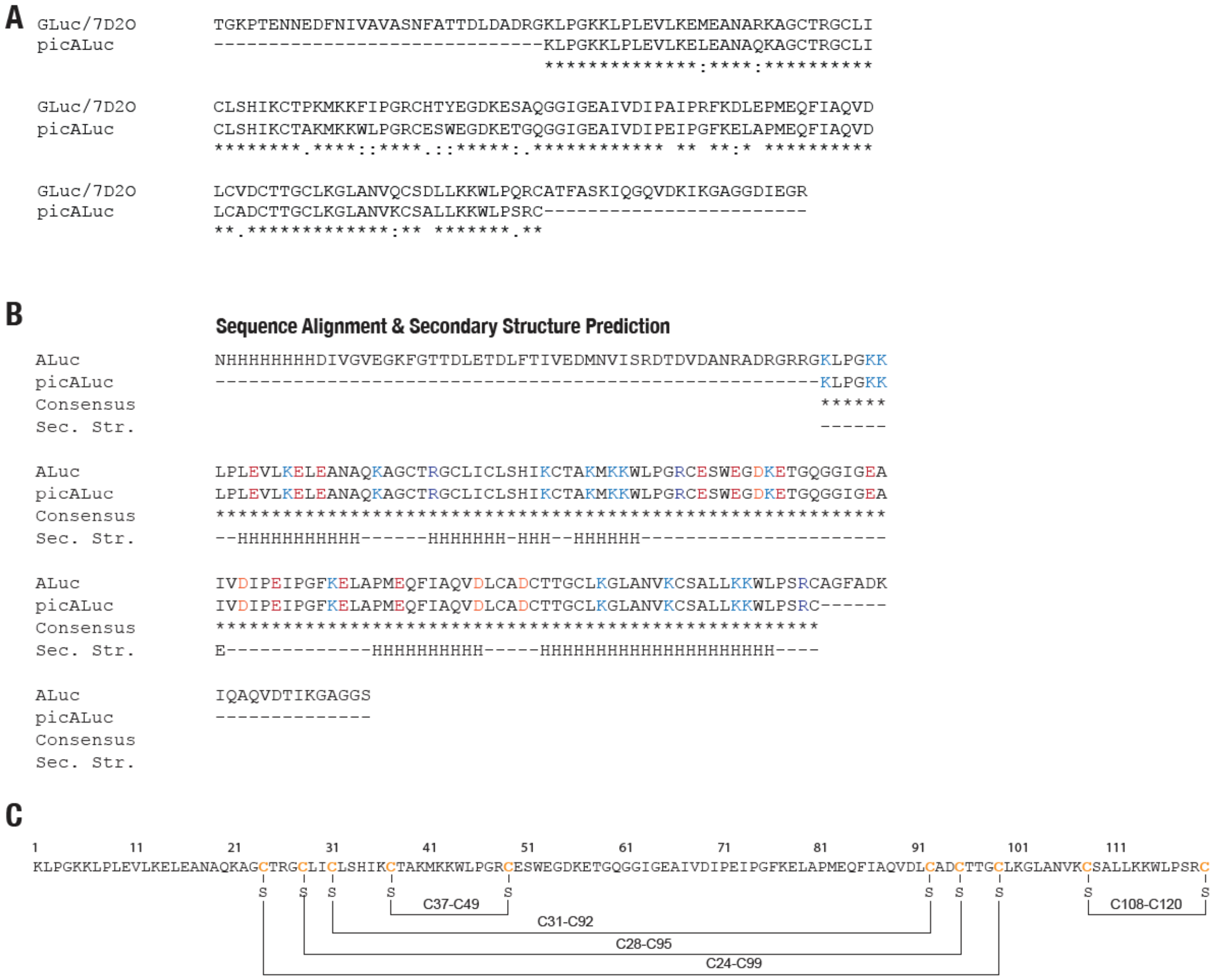

Figure S1. Sequence alignments and disulfide bridges in picALuc. (**A**) Sequence alignment of *Gaussia* luciferase (GLuc) and picALuc. (**B**) Sequence alignment of picALuc and ALuc. Positively and negatively charged residues are highlighted in blue (light blue, Lys; deep blue, Arg) and red (light red, Asp; deep red, Glu), respectively. Secondary structure prediction is shown in the lower panel. (**C**) Amino acid sequence of picALuc highlighting all disulfide bridges observed in the structural model of picALuc generated from the NMR structure of GLuc [PDB: 7D2O] [1].

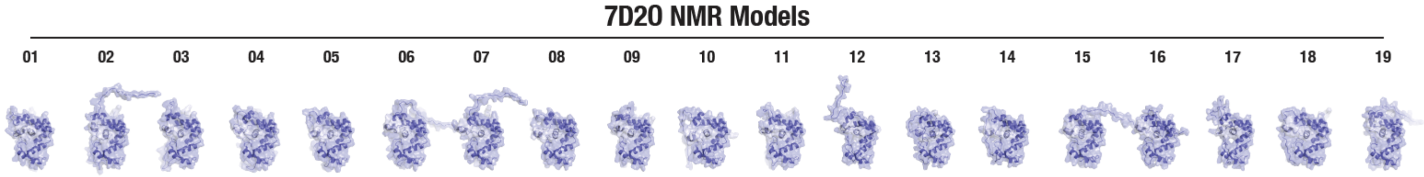

Figure S2. GLuc structural flexibility. Surface and cartoon representation of GLuc NMR models (conformers; [PDB: 7D2O] [1]) revealing flexibility of the N-terminal region of the protein.

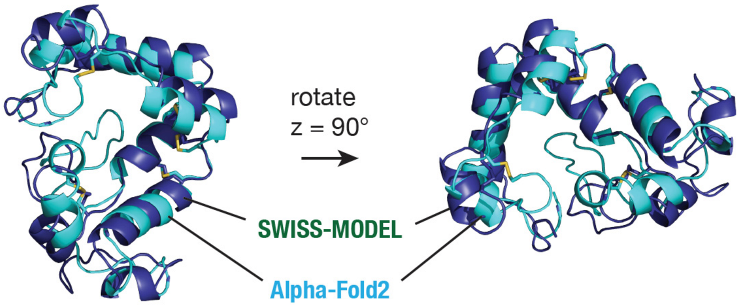

Figure S3. Comparison of picALuc structural models generated using SWISS-MODEL and Alpha-Fold2. Cartoon representation of picALuc structural models generated using SWISS-MODEL (blue) and Alpha-Fold2 [2] (cyan) with an RMSD of 3.276 Å.

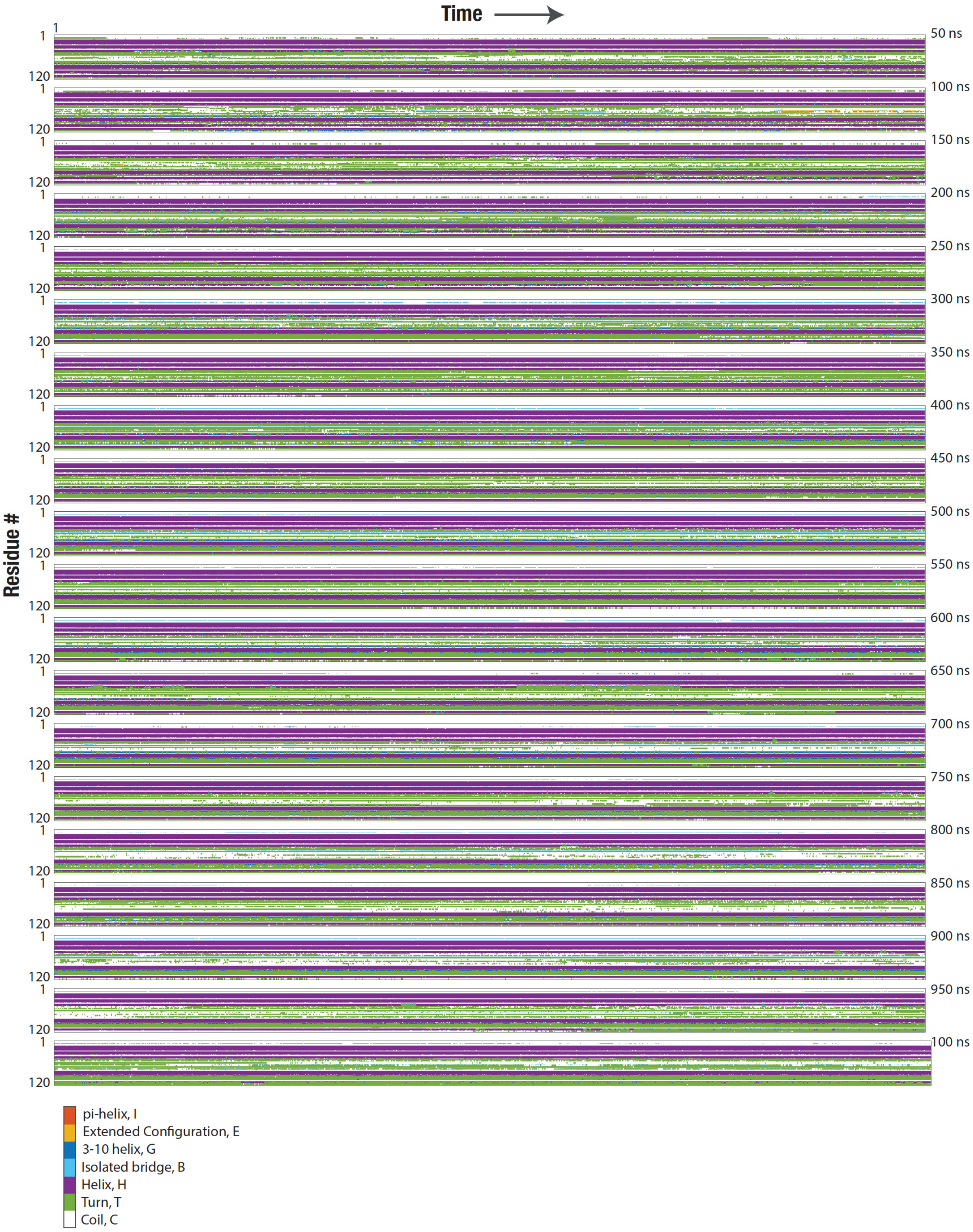

Figure S4. Secondary structure of picALuc from GaMD simulation. Schematic representation of secondary structure of picALuc over the course of course of 1 µs of GaMD simulation.

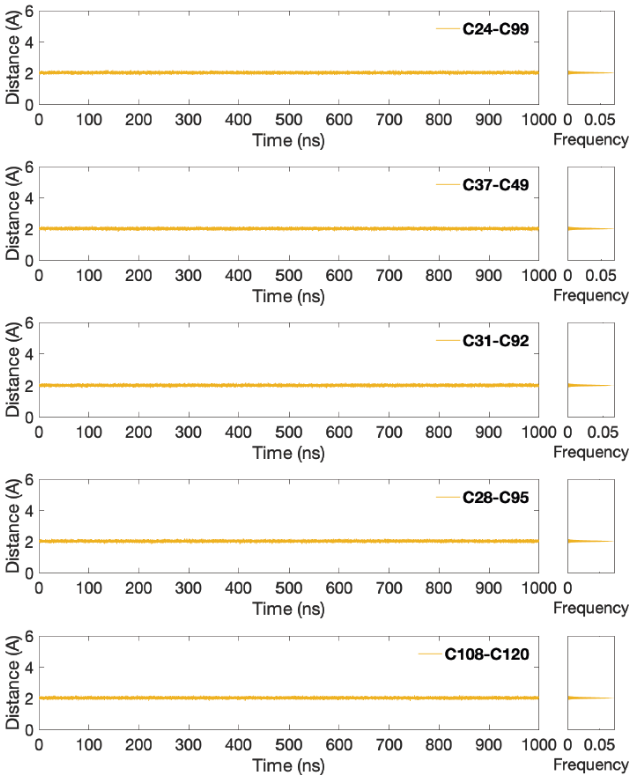

Figure S5. Stability of disulfide bridges in picALuc. Graphs showing S atom distances between the indicated disulfide forming Cys residues of picALuc over the course of course of 1 µs of GaMD simulation.

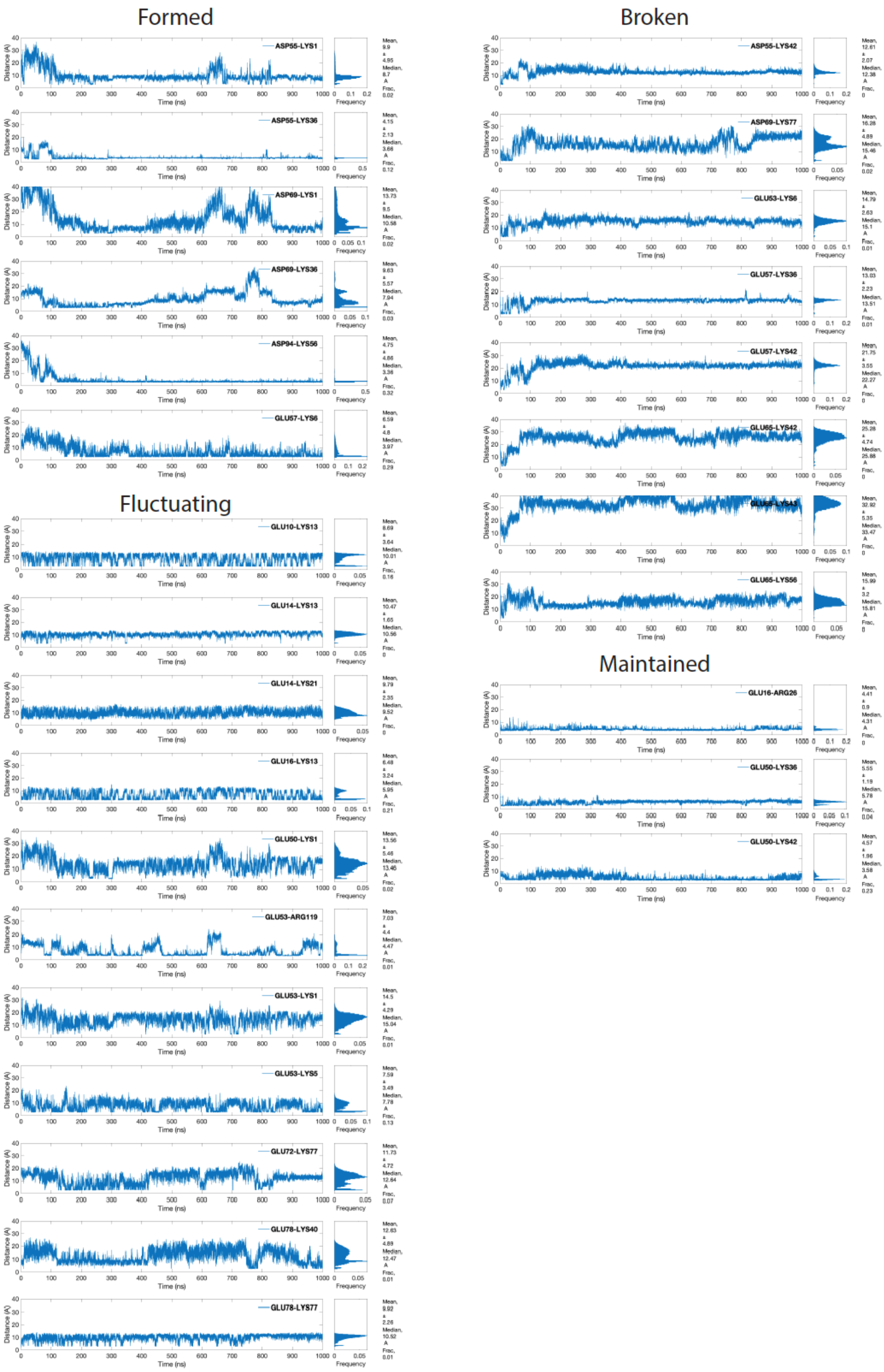

Figure S6. Salt bridges observed in picALuc. Graphs showing distances between O and N atoms of the salt bridge forming indicated residues over the course of course of 1 µs of GaMD simulation as determined using the Salt Bridges Plugin available in the VMD software (version 1.9.3) [3]. The interactions have been grouped as those that are formed during the simulation, those that are broken during the simulation, those that fluctuate during the simulation and those that are maintained during the course of the simulation.

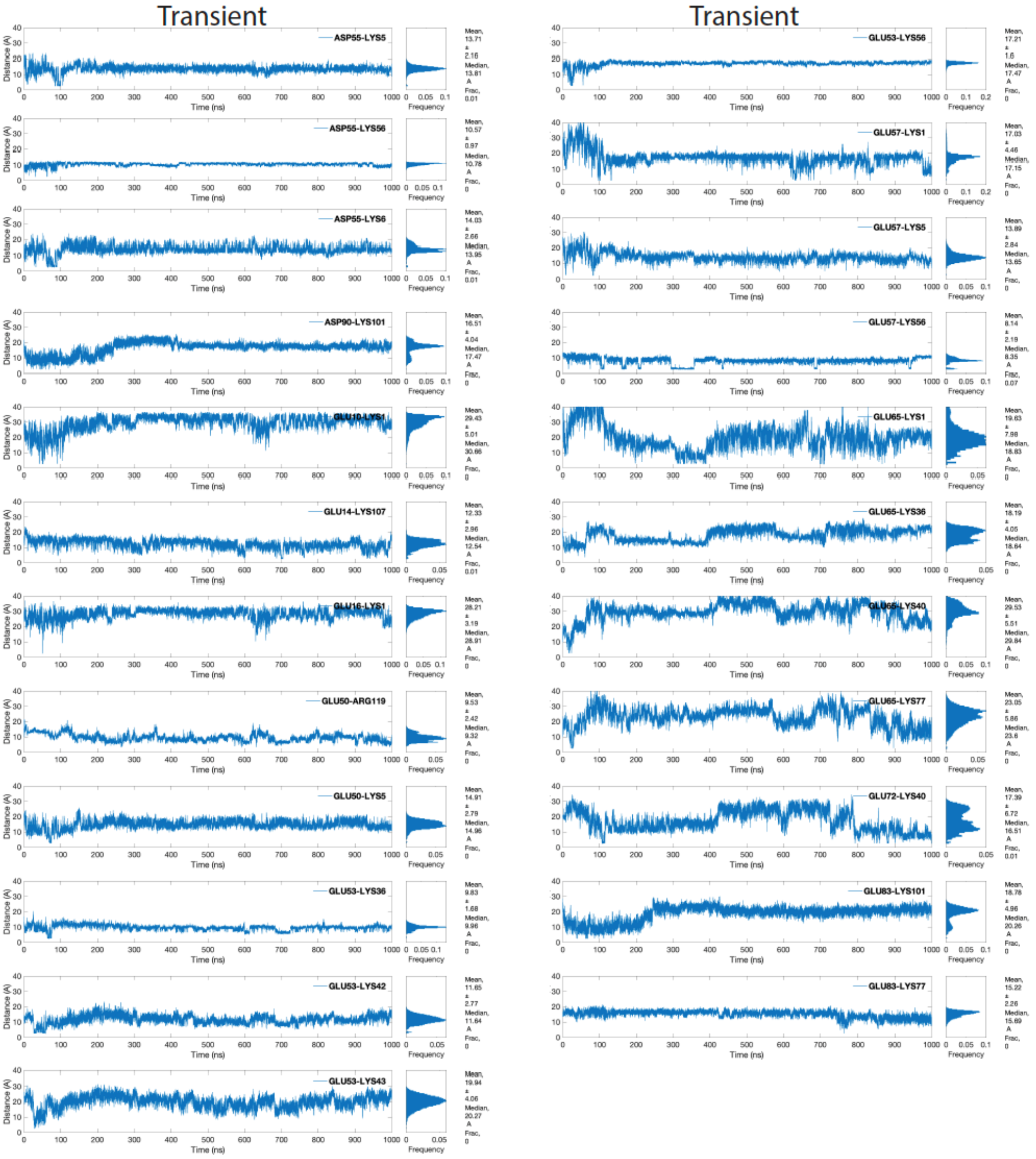

Figure S7. Transient salt bridges observed in picALuc. Graphs showing distances between O and N atoms of indicated residues that form transient salt bridges over the course of course of 1 µs of GaMD simulation.

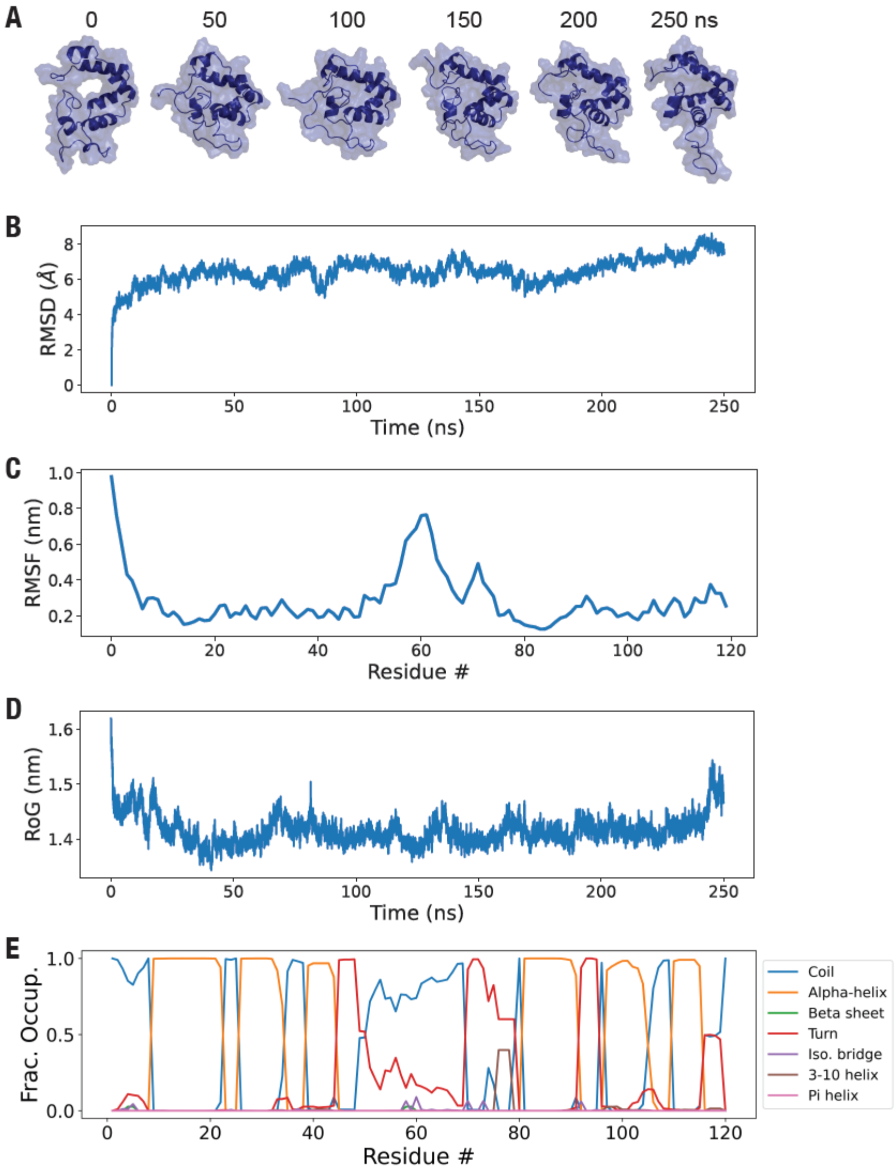

Figure S8. Conventional MD simulation of picALuc. (**A**) Cartoon and surface representation of picALuc over 250 ns of conventional MD simulation showing rapid structural evolution of the protein. (**B** – **D**) Graphs showing Ca atom RMSD (**B**), RMSF (**C**) and RoG (**D**) values of picALuc obtained from conventional simulation. (**E**) Graph showing frequency of various secondary structural elements against picALuc residues.

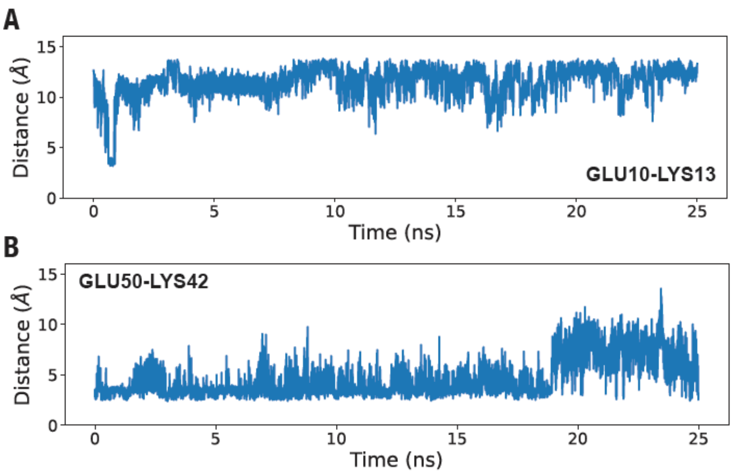

Figure S9. Salt bridges observed in conventional MD simulation of picALuc. Graphs showing distances between O and N atoms of indicated residues in picALuc that form salt bridge interaction over the course of 250 ns of conventional MD simulation.

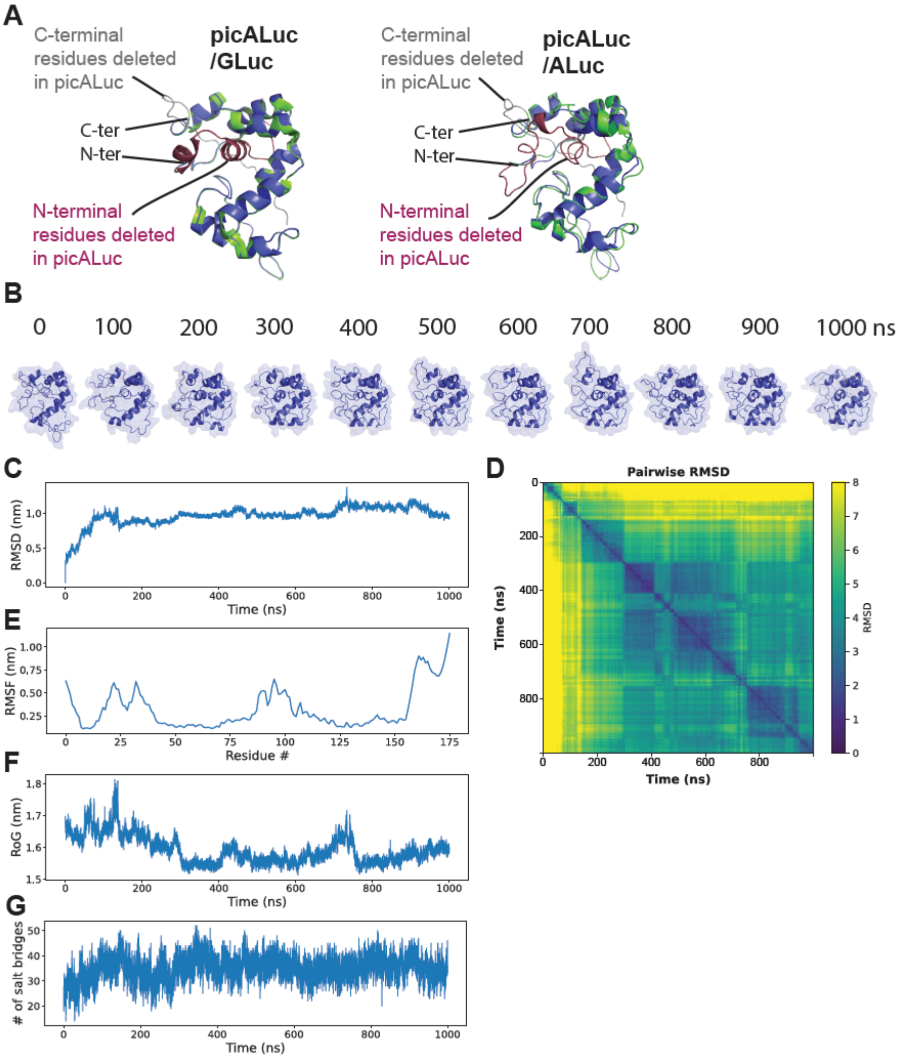

Figure S10. GaMD simulation of ALuc. (**A**) Cartoon representation showing comparison of the picALuc (blue) structural model with GLuc structure (green; PDB: 7D2O [1]; left panel) and ALuc

(green) structural model (right panel) with the N- and C-terminal residues deleted in picALuc. Note that while the C-terminal residues deleted in picALuc are external to the protein core, the N-terminal residues deleted in picALuc are internal to the protein core suggestive of non-trivial impact of the deletion of N-terminal residues. N- and C-termini of picALuc are indicated with lines. (**B**) Cartoon and surface representation of ALuc structure captured every 100 ns over the course of 1 µs GaMD simulation. (**C** – **E**) Graphs showing Ca atom RMSD (**C**), pairwise RMSD (**D**) and RMSF (**E**) values of ALuc obtained from the GaMD simulation. (**F**,**G**) Graphs showing radius of gyration (RoG) (**F**) and number of salt bridge interactions (**G**) of ALuc over the course of 1 µs GaMD simulation.

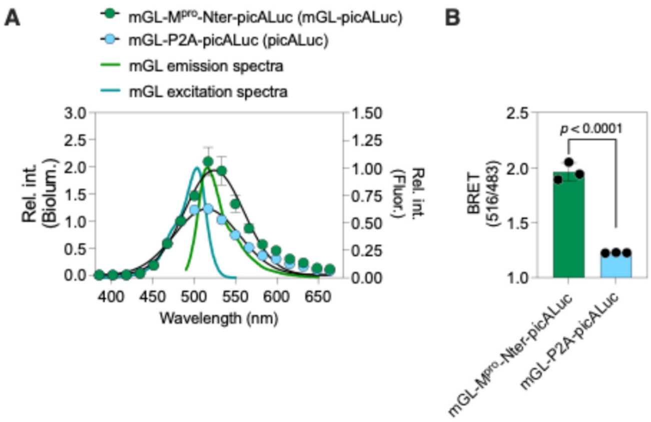

Figure S11. Bioluminescence characterization of mGL-picALuc constructs. (**A**) Graph showing bioluminescence spectra of mGL-Mpro-Nter-picALuc and mGL-P2A-picALuc obtained from live cells. Fluorescence excitation and emission spectra of mGL (obtained from fpbase.org) are also included for comparison. (**B**) Graph showing BRET[4, 5] (ratio of emissions at 516 and 483 nm) of the indicated proteins obtained from live cells.

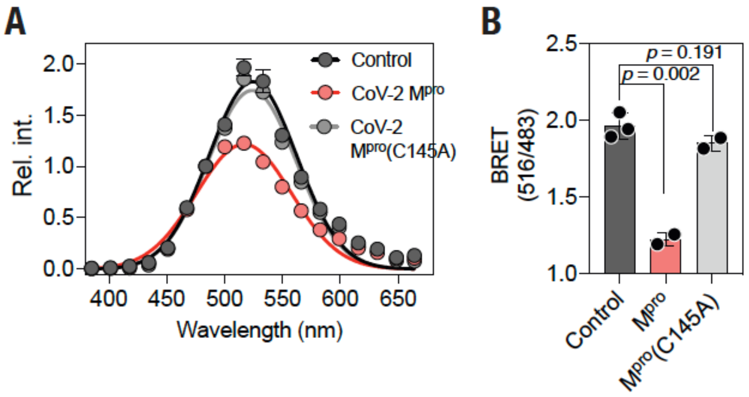

Figure S12. SARS-CoV-2 Mpro-mediated cleavage of mGL-Mpro-Nter-picALuc biosensor. (**A**) Graph showing bioluminescence spectra of mGL-picALuc obtained from live cells in the absence or presence of either WT or C145A mutant SARS-CoV-2 Mpro. (**B**) Graph showing BRET[4-6] (ratio of emis-

sions at 516 and 483 nm) of mGL-picALuc obtained from live cells in the absence or presence of either WT or C145A mutant SARS-CoV-2 M^Pro^.

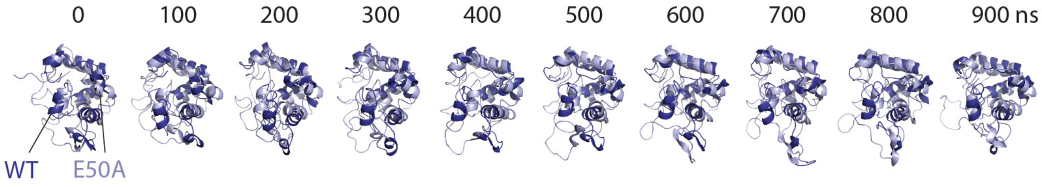

Figure S13. Structural dynamics of WT and E50A mutant picALuc. Cartoon representation of the WT and E50A mutant picALuc obtained from GaMD simulations.

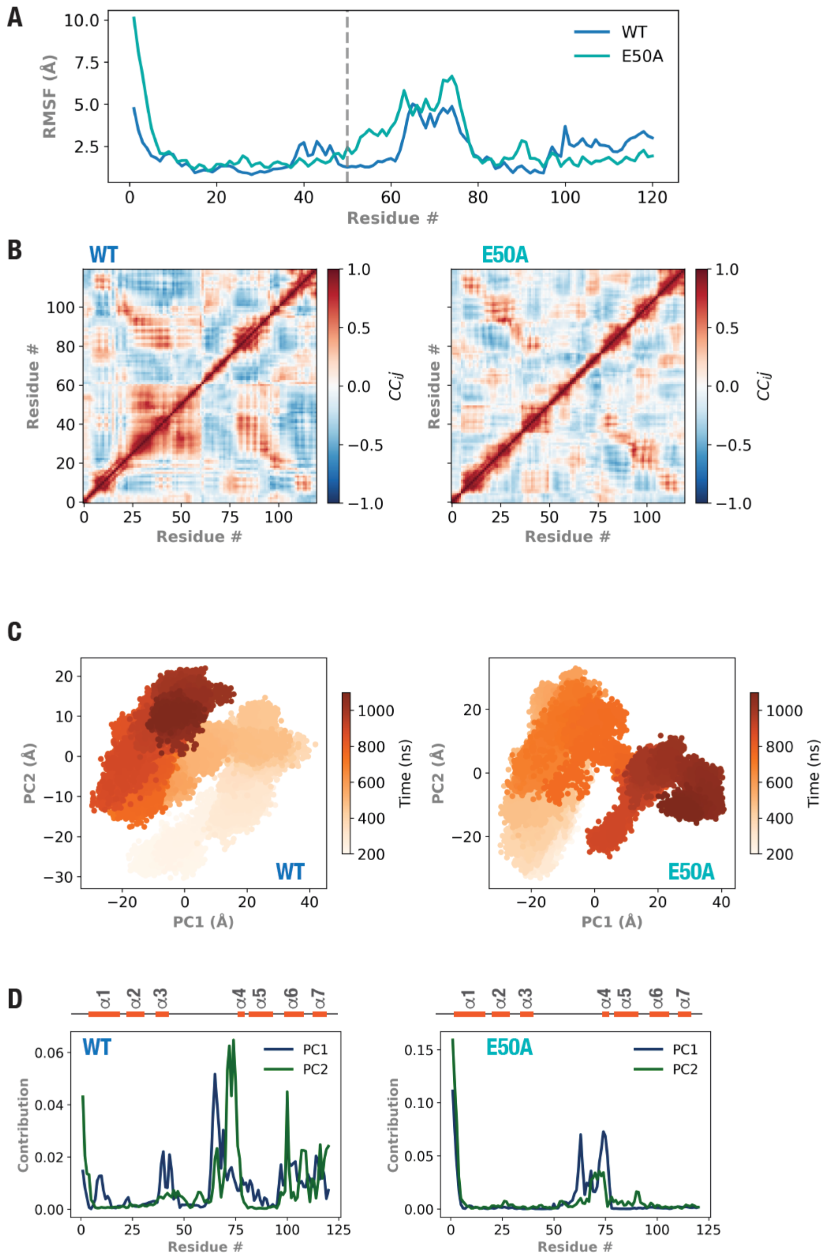

Figure S14. Collective dynamics of WT and E50A mutant picALuc obtained from from the last 900 ns of GaMD simulations. (**A**) Graph showing Ca atom RMSF values of the WT and E50A mutant

picALuc obtained from the last 900 ns of GaMD simulations. (**B**) Plots showing DCC of residues in the WT (left panel) and E50A mutant (right panel) picALuc determined from the last 900 ns of GaMD trajectories of each protein. (**C**) Graphs showing principal components 1 and 2 (PC1 and PC2) of the WT (left panel) and E50A mutant (right panel) determined from the last 900 ns of GaMD trajectories of each protein. Color bar, time (ns). (**D**) Graphs showing contribution of individual residues to the PC1 and PC2 in the WT (left panel) and the E50A mutant (right panel) picALuc simulations determined from the last 900 ns of GaMD trajectories of each protein. Location of residue number 50 is highlighted using a red line, respectively in each graph.

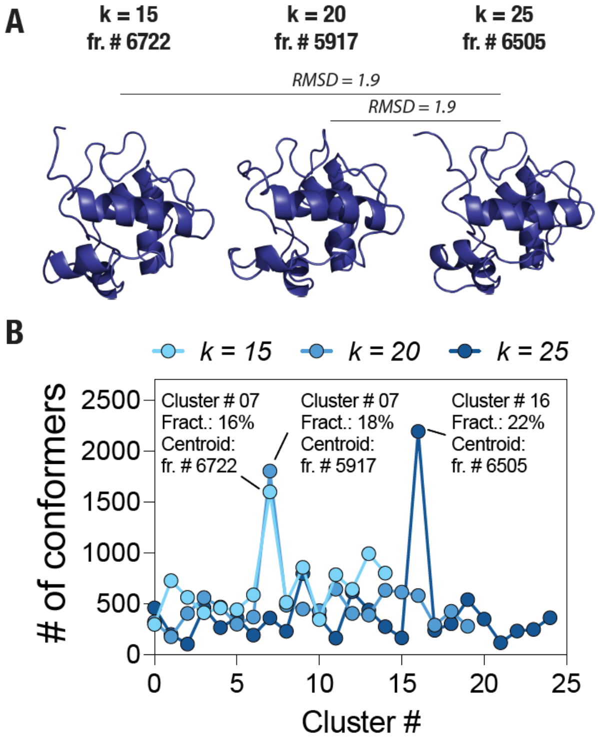

Figure S15. Clustering analysis of picALuc conformers obtained from GaMD simulation trajectory. (**A**) Cartoon representation of the picALuc conformers obtained from k-means clustering of the 1 µs GaMD simulation trajectory at a step size of 5 with the indicated number of clusters (k). Conformers shown are the centroids of the clusters containing maximum number of conformers. RMSD values shown were obtained from alignment of the k = 15 and 20 centroid conformers with the k = 25 centroid conformer. (**B**) Graph showing number of conformers per cluster obtained from k-means clustering of the 1 µs GaMD simulation trajectory at a step size of 5.

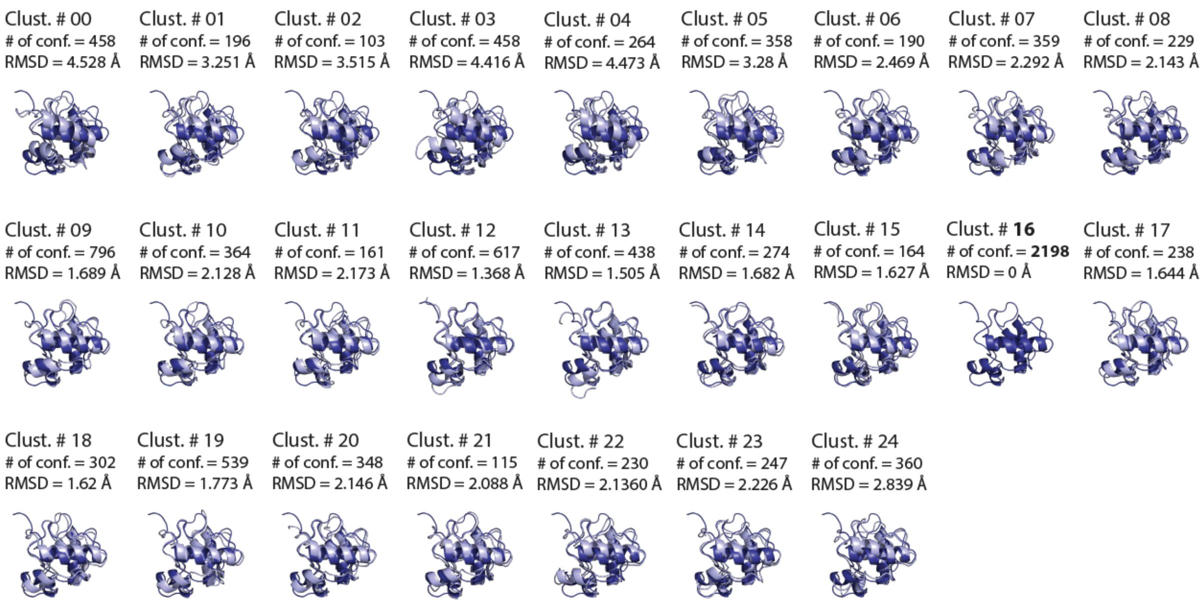

Figure S16. Conformational analysis of picALuc obtained from GaMD simulation. Cartoon representation of the centroid conformer of each of the 25 clusters obtained from k-means clustering of the picALuc 1 µs GaMD simulation trajectory at a step size of 5 (in comparison to the centroid conformer of cluster # 16 containing the highest number of conformers). Number of conformers and RMSD with the centroid of the cluster # 16 are shown for each cluster.

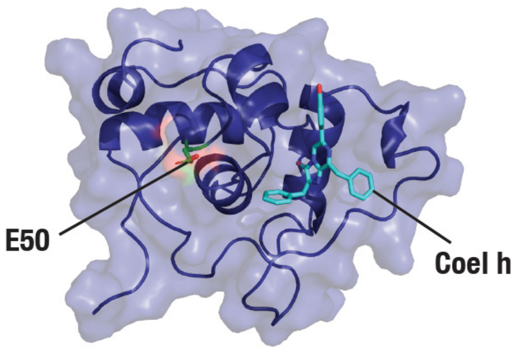

Figure S17. Docking of substrate on picALuc structural model. Cartoon and surface representation of picALuc showing docked substrate, coelenterazine h.

References

1. Wu, N., et al., *Solution structure of Gaussia Luciferase with five disulfide bonds and identification of a putative coelenterazine binding cavity by heteronuclear NMR.* Sci Rep, 2020. **10**(1): p. 20069.
2. Jumper, J., et al., *Highly accurate protein structure prediction with AlphaFold.* Nature, 2021. **596**(7873): p. 583-589.
3. Humphrey, W., A. Dalke, and K. Schulten, *VMD: visual molecular dynamics.* J Mol Graph, 1996. **14**(1): p. 33-8, 27-8.
4. Biswas, K.H. and S.S. Visweswariah, *Distinct allostery induced in the cyclic GMP-binding, cyclic GMP-specific phosphodiesterase (PDE5) by cyclic GMP, sildenafil, and metal ions.* Journal of Biological Chemistry, 2011. **286**(10): p. 8545-54.
5. Biswas, K.H., S. Sopory, and S.S. Visweswariah, *The GAF domain of the cGMP-binding, cGMP-specific phosphodiesterase (PDE5) is a sensor and a sink for cGMP.* Biochemistry, 2008. **47**(11): p. 3534-43.
6. Geethakumari, A.M., et al., *A genetically encoded BRET-based SARS-CoV-2 M(pro) protease activity sensor.* Commun Chem, 2022. **5**(1): p. 117.
